# SIRT3 deficiency decreases oxidative metabolism capacity but increases lifespan in male mice under caloric restriction

**DOI:** 10.1111/acel.13721

**Published:** 2022-10-05

**Authors:** Rashpal S. Dhillon, Yiming (Amy) Qin, Paul R. van Ginkel, Vivian X. Fu, James M. Vann, Alexis J. Lawton, Cara L. Green, Fúlvia B. Manchado‐Gobatto, Claudio A. Gobatto, Dudley W. Lamming, Tomas A. Prolla, John M. Denu

**Affiliations:** ^1^ Department of Biomolecular Chemistry University of Wisconsin‐Madison Madison Wisconsin USA; ^2^ Wisconsin Institute for Discovery University of Wisconsin‐Madison Madison Wisconsin USA; ^3^ Interdepartmental Graduate Program in Nutritional Sciences University of Wisconsin‐Madison Madison Wisconsin USA; ^4^ Department of Genetics and Medical Genetics University of Wisconsin‐Madison Madison Wisconsin USA; ^5^ Department of Medicine, SMPH University of Wisconsin‐Madison Madison Wisconsin USA; ^6^ William S. Middleton Memorial Veterans Hospital Madison Wisconsin USA; ^7^ Laboratory of Applied Sport Physiology, School of Applied Sciences University of Campinas Limeira Brazil

**Keywords:** aerobic fitness, calorie restriction, fatty acid oxidation, fuel switching, lifespan, mitochondrial acetylation, mitochondrial respiration, sirtuins

## Abstract

Mitochondrial NAD^+^‐dependent protein deacetylase Sirtuin3 (SIRT3) has been proposed to mediate calorie restriction (CR)‐dependent metabolic regulation and lifespan extension. Here, we investigated the role of SIRT3 in CR‐mediated longevity, mitochondrial function, and aerobic fitness. We report that SIRT3 is required for whole‐body aerobic capacity but is dispensable for CR‐dependent lifespan extension. Under CR, loss of SIRT3 (*Sirt3* ^*−/−*^) yielded a longer overall and maximum lifespan as compared to *Sirt3* ^*+/+*^ mice. This unexpected lifespan extension was associated with altered mitochondrial protein acetylation in oxidative metabolic pathways, reduced mitochondrial respiration, and reduced aerobic exercise capacity. Also, *Sirt3* ^*−/−*^CR mice exhibit lower spontaneous activity and a trend favoring fatty acid oxidation during the postprandial period. This study shows the uncoupling of lifespan and healthspan parameters (aerobic fitness and spontaneous activity) and provides new insights into SIRT3 function in CR adaptation, fuel utilization, and aging.

## Graphical abstract

This study shows that lifespan and healthspan parameters can be uncoupled and that the mitochondrial protein SIRT3 is a modulator of fuel utilization, physical activity, and aging.

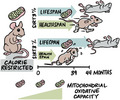

**Abbreviations**
ANCOVA: analysis of covariance
ANOVA: analysis of variance
BCAA: branched‐chain amino acids
CD: control diet
CR: calorie restriction
ETC: electron transport chain
FAO: fatty acid oxidation
GSH: glutathione
MS: mass spectrometry
NNT: nucleotide transhydrogenase
OCR: oxygen consumption rate
RER: respiratory exchange ratio
SEM: standard error of the mean
SIRT3: sirtuin3
SPA: spontaneous physical activity

## 1 INTRODUCTION

Incidence of non‐communicable diseases, such as cardiovascular disease, neurodegeneration, and cancer, increases significantly with age (Balasubramanian et al., 2017). Calorie restriction (CR) robustly delays age‐related diseases and extends both health and lifespan in diverse species (Fontana & Partridge, 2015; Mattison et al., 2017). Mitochondria, the center of cellular oxidative metabolism, are vital to cellular health, and mitochondrial dysfunction has been associated with accelerated aging (Jang et al., 2018; Srivastava, 2017). One of the hallmarks of CR is the preservation of mitochondrial function through reducing oxidative stress, enhancing fuel utilization, and maintaining mitochondrial dynamics and integrity (Bruss et al., 2010; Jang et al., 2018; Lanza et al., 2012; Merry, 2004). Previous studies have proposed that Sirtuin3 (SIRT3)‐dependent deacetylation may play a major role in modulating mitochondria under CR (Hallows et al., 2011; Qiu et al., 2010; Someya et al., 2010).

Reversible *N* ^ε^‐lysine acetylation is a prominent post‐translational modification enriched in mitochondria, with more than 60% of all mitochondrial proteins having at least one acetylated lysine site identified in proteomic studies (Baeza et al., 2016; Liu et al., 2014). Among well‐characterized examples, mitochondrial acetylation mostly inhibits enzymatic activity, slows down metabolic pathways, and leads to mitochondrial dysfunction (Hirschey et al., 2010; Still et al., 2013; Vassilopoulos et al., 2014) which has been correlated with diabetes, heart failure, and age‐related cellular impairment (Ansari et al., 2017; Fukushima & Lopaschuk, 2016; Horton et al., 2016). SIRT3 is the predominant NAD^+^‐dependent deacetylase in mitochondria, whose deficiency leads to significant hyperacetylation in mitochondria of various tissues (Dittenhafer‐Reed et al., 2015). SIRT3 level is diet‐sensitive, and its expression increases under fasting and CR (Palacios et al., 2009; Schwer et al., 2009). We have reported SIRT3‐dependent deacetylation of mitochondrial proteins in CR mice and demonstrated that SIRT3 is essential for the prevention of age‐related hearing loss in mice fed a CR diet (Someya et al., 2010). These observations have fueled the speculation that SIRT3‐dependent control of acetylation may be crucial for CR‐induced modulation of aging and lifespan extension. Nonetheless, direct evaluation of the contribution by SIRT3 to CR‐dependent lifespan extension and mitochondrial performance during aging is lacking.

Here, using male *Sirt3* ^*+/+*^ (WT) and *Sirt3* ^*−/−*^ mice, both in a C57BL/6NJ × C57BL/6J NNT wildtype genetic background, treated with either a control diet (CD, under moderate CR) or CR diet (25% reduction from CD), we found that (1) SIRT3 is essential for whole‐body aerobic fitness as assessed by critical velocity, but is not required to mediate CR‐dependent longevity, (2) loss of SIRT3 reduces the net OXPHOS respiration when glucose‐derived metabolites are utilized, and (3) SIRT3 ablation appears to favor fuel switching from glucose to fatty acids during the postprandial state. We also show that SIRT3 is required for CR‐induced increases in spontaneous physical activity (SPA). Collectively, these data reveal that SIRT3 deficiency under CR increases lifespan beyond CR alone and that this condition is associated with lower aerobic fitness, lower spontaneous activity, and altered mitochondrial metabolism favoring fatty acid metabolism.

## 2 RESULTS

### 2.1 SIRT3 ablation under CR extends lifespan and has minimal impact on mitochondrial integrity and ROS detoxification

**FIGURE 1:**
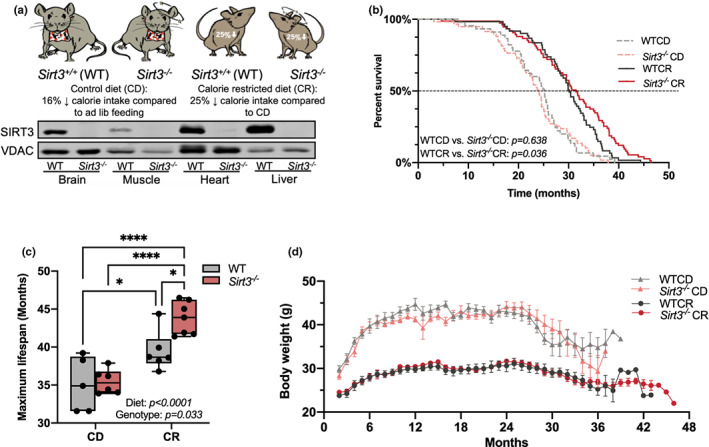
SIRT3 ablation under CR extends lifespan. (a) Mouse models in the current study. See “Methods” section for detailed feeding regimen. (b) Kaplan–Meier survival curves for WTCD (n = 45), WTCR (n = 61), Sirt3^−/−^CD (n = 59), and Sirt3^−/−^CR mice (n = 75). The dashed line (50% survival probability) indicates the median lifespan. Log‐rank test was used to calculate statistical significance of lifespan among treatment groups. See Table S1a for detailed statistics and Table S3 for individual mouse lifespan. (c) Maximum lifespan is represented by the average lifespan of the top 10% longest‐lived mice for each group, WTCD (n = 5), WTCR (n = 6), Sirt3^−/−^CD (n = 6), and Sirt3^−/−^CR mice (n = 7). Data were analyzed by two‐way ANOVA followed by multiple comparisons test. *p* value reported for each comparison is corrected by Tukey's test. Results are plotted as mean with min. to max. range. *p ≤ 0.05;   ****p ≤ 0.0001. We also confirmed the statistical significance of maximum lifespan between WTCR and Sirt3^−/−^CR using Boschloo's test (Wang‐Allison, Table S1b). (d) Lifetime body weight for mice used in lifespan study is shown in Figure 1b. ANOVA, analysis of variance; CD, control diet; CR, calorie restriction; SIRT3, sirtuin3

To investigate the role of SIRT3 in CR‐dependent metabolism during aging, we established whole‐body *Sirt3* ^*−/−*^ mouse models in the *Nnt* ^*+/+*^ background by crossing C57BL/J (*Nnt* ^*−/−*^ *) Sirt3* ^*−/−*^ mice with C57BL/6NJ (*Nnt* ^*+/+*^) *Sirt3* ^*+/+*^ mice. This is noteworthy in that many prior aging and SIRT3 studies employed C57BL/6J mice that lack functional mitochondrial nicotinamide nucleotide transhydrogenase (NNT). With increasing evidence demonstrating the importance of NNT in redox control and metabolism (Ronchi et al., 2016; Smith et al., 2020), we generated and used C57BL/6NJ × C57BL/6J background, NNT wild‐type *Sirt3* ^*+/+*^ and NNT wild‐type *Sirt3* ^*−/−*^ mice in the current study. From 2 months of age until death, male WT and *Sirt3* ^*−/−*^ mice were maintained on a fixed calorie CD (89 kcal/week) or CR diet (67 kcal/week, 25% less calorie intake than CD‐fed mice) (Figure 1a). Notably, CD mice in the current study were fed 16% less calories compared to ad libitum mice (105.8 kcal/week) to reduce obesity and related metabolic complications (see Methods for detailed diet design; Pugh et al., 1999). Both CD and CR groups were fed three times/week (Monday, Wednesday, and Friday at ~7 AM). CR mice generally finished food within 12–24 h, and CD mice finished food within 24–36 h. No genotype‐dependent food consumption difference was observed. Thus, all study groups were under an intermittent fasting protocol with CR mice experiencing a slightly longer fasting time. Body weight averages increased until ~12 months of age and were maintained until ~27 months of age, after which average weight values declined in both CR and CD groups (Figure 1d). At peak weights, body weights were approximately 25% lower in CR mice (Figure 1d). Since food intake was fixed, variations in body weight in animals during their lifespan likely resulted in alterations in food consumption relative to body weight in individual animals at different ages.

Kaplan–Meier survival curves (Figure 1b; Table S1a) revealed that as expected, CR significantly extended the average (WTCD: 24.1 months, WTCR: 29.7 months), median (50% survival, WTCD: 25.2 months, WTCR: 30.1 months), and maximum lifespan (WTCD: 35.1 months, WTCR: 39.4 months, calculated as the average lifespan of the top 10% longest‐lived mice) of WT mice (Figure 1c). *Sirt3* ^*−/−*^ mice exhibited a similar average (WTCD vs. *Sirt3* ^*−/−*^CD: 24.1 vs. 23.5 months, WTCR vs. *Sirt3* ^*−/−*^CR: 29.7 vs. 31.0 months), median lifespan (WTCD vs. *Sirt3* ^*−/−*^CD: 25.2 vs. 24.0 months, WTCR vs. *Sirt3* ^*−/−*^CR: 30.1 vs. 31.5 months) as their diet‐matched counterparts under both CD and CR, but unexpectedly *Sirt3* ^*−/−*^CR mice showed an overall increase in lifespan as compared to WTCR mice (*p* = 0.036, Table S1a). Remarkably, *Sirt3* ^*−/−*^CR mice displayed a 10% increase in maximum lifespan relative to WTCR (WTCR vs. *Sirt3* ^*−/−*^CR: 39.4 vs. 43.8 months, *p* = 0.03, Figure 1c. Confirmed with Boschloo's test, *p* = 0.04 at 90% percentile for WTCR versus *Sirt3* ^*−/−*^CR, Table S1b). These results show that SIRT3 is not required for the normal life extension benefit of CR and that SIRT3 ablation further extends CR‐mediated longevity. Interestingly, the *Sirt3* ^*−/−*^‐dependent overall lifespan extension benefit was not noticeable until a later age, as the survival curves of WTCR and *Sirt3* ^*−/−*^CR mice diverge after the median lifespan of these groups (Figure 1b) and a significant change in 75% percentile in WTCR versus *Sirt3* ^*−/−*^CR was observed (*p* = 0.03, Table S1b). Lifetime body weight (Figure 1d) and body composition at 25 months of age (Figure S1a–c) showed a diet but not a SIRT3 dependence. Together, these observations revealed an unexpected boost of maximum lifespan in *Sirt3* ^*−/−*^ mice under CR.

Several reports have highlighted the ability of SIRT3 to preserve mitochondrial integrity and suppress oxidative stress through protein deacetylation (Bause & Haigis, 2013; Kim et al., 2010; Meng et al., 2019; Qiu et al., 2010) including work demonstrating that CR prevents age‐related hearing loss through SIRT3‐dependent mechanism involving IDH2 activation (Someya et al., 2010; Yu et al., 2012). Recently, Benigni et al. (2019) observed that shortened lifespan in standard chow‐fed *Sirt3* ^*−/−*^ mice is associated with altered cardiac mitochondrial morphology. Given the unexpected longevity in *Sirt3* ^*−/−*^CR mice, we investigated whether mitochondrial integrity as examined by transmission electron microscopy was affected at 25 months of age. No morphological differences in gastrocnemius and heart mitochondria were observed between genotypes nor diets (Figure S1d). Similarly, citrate synthase activity, a surrogate marker of mitochondrial density, and its protein expression in the brain, gastrocnemius, heart, and liver were comparable between groups (Figure S1e,f). Moreover, there was no significant link between SIRT3 expression (Figure S1g) and mitochondrial DNA content (mtDNA:nDNA, Figure S1h). NADPH/NADP^+^ and GSH/GSSG ratios were comparable between *Sirt3* ^*−/−*^ and WT animals under both diets (Figure S1i,j) in the liver and heart. In summary, these data indicate that loss of SIRT3 has no significant impact on mitochondrial integrity and markers of ROS detoxification in aged C57BL/6NJ x C57BL/6J *Nnt* ^*+/+*^ mice.

### 2.2 SIRT3 opposes hyperacetylation of specific target proteins under CR during aging

Consistent with the role of SIRT3 as a mitochondrial deacetylase, immunoblotting of total mitochondrial protein acetylation in liver from 25‐month‐old mice displayed an increase with SIRT3 ablation and a combined effect of CR and loss of SIRT3 (Figure S2a). To identify the lysine sites and proteins showing altered acetylation as a function of age, genotype, and diet, we leveraged our recently developed quantitative mass spectrometry workflow (Baeza et al., 2020) (Figure S2b). Site‐specific acetylation stoichiometry from enriched liver mitochondrial fractions was determined in 5‐ and 25‐month‐old WTCD, WTCR, *Sirt3* ^*−/−*^CD, and *Sirt3* ^*−/−*^CR mice. From 1854 unique mitochondrial acetyl‐lysine sites identified (≤1% false discovery rate), 941 sites on 329 proteins (MitoCarta 2.0, Calvo et al. (2016), Table S2a) were quantified in all eight treatment groups, with acetylation stoichiometry ranging from less than 1% to 99% and a median stoichiometry of ~8.3% among quantified sites.

**FIGURE 2:**
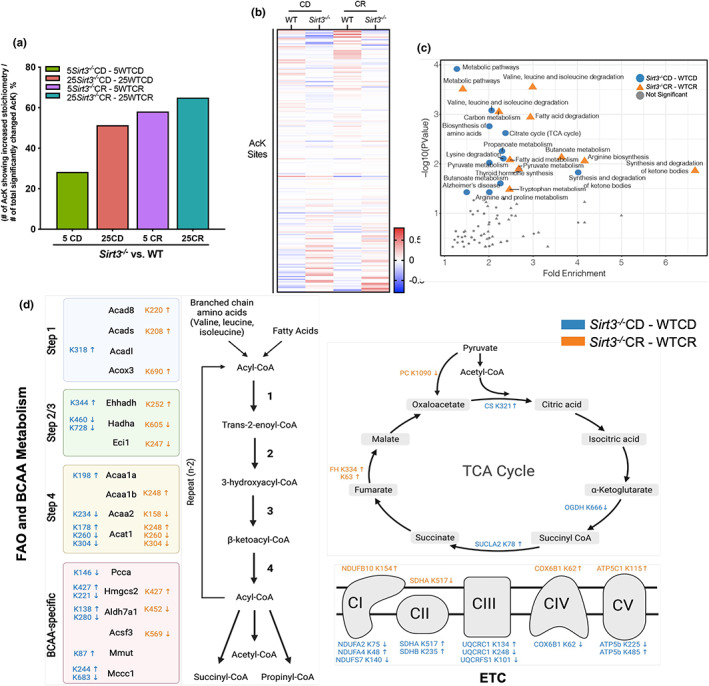
SIRT3 opposes hyperacetylation of its targets under CR during aging. (a–d) Liver mitochondrial acetylome analysis using stoichiometry‐based MS quantification. See Table S2 for all identified acetyl‐lysine sites and statistical analysis, n = 4 per group. (a) Percentage of significantly changed acetyl‐lysine residues that show increased stoichiometry due to Sirt3^−/−^ status, calculated by (the number of acetyl‐lysine sites showing increased stoichiometry)/(the number of significantly changed acetyl‐lysine sites, *p* < 0.05 ×100%. 5: 5 months old; 25: 25 months old. (b) Heat map of significantly changed lysine sites *p* < 0.05 in 25 month‐old mice that are a response to loss of SIRT3. Plotted sites are significantly changed (*p* < 0.1) in either Sirt3^−/−^CD versus WTCD or Sirt3^−/−^CR versus WTCR comparison. Values are colored based on relative acetylation stoichiometry, normalized to the median value of each site in all four groups, scaling ranging from ‐0.8 to 0.8 (×100%). (c) Functional cluster analysis of KEGG pathways (DAVID 6.8). Significantly enriched (−log10(*p* value) >1.5) pathways are indicated, with 25 month‐old Sirt3^−/−^CD versus WTCD in orange and 25 month‐old Sirt3^−/−^CR versus WTCR in blue. (d) Acetylation sites in FAO and BACC metabolism, TCA cycle, and ETC that displayed larger than 5% stoichiometry (*p* < 0.1) for 25 month‐old Sirt3^−/−^CD versus WTCD (orange colored) and 25 month‐old Sirt3^−/−^CR versus WTCR comparison (blue colored). CD, control diet; CR, calorie restriction; MS, mass spectrometry; SIRT3, sirtuin3

The effects of diet, age, and genotype were parsed out (Figure S2c–f) to reveal the relative number of changed acetylation sites (density) as a function of change in stoichiometry for those conditions. An age‐mediated increase in acetylation stoichiometry in mice fed a CR diet was evident. Both young WT and *Sirt3* ^*−/−*^CR mice showed less acetylation alteration and accumulation, suggesting that aged mice are more prone to CR‐induced hyperacetylation (Figure S2d). Additionally, the stoichiometry disparities between aged and young mice were more notable when the mice are *Sirt3* ^*−/−*^ and CR fed, indicating that both CR and *Sirt3* ^*−/−*^ amplify the age‐dependent acetylation alteration (Figure S2e). Lastly, SIRT3‐dependent acetylation changes are most significant in aged, CR‐treated animals (Figure S2f), with the 25‐month *Sirt3* ^*−/−*^CR versus 25‐month WTCR comparison showing the largest fraction of increased acetylation (Figure 2a) among the various groups. In sum, these results indicate that CR and loss of SIRT3 individually and cooperatively contribute to the global increase in acetylation at old age.

To reveal specific site‐level acetylation changes across the various conditions, we plotted the relative stoichiometry of significantly changed sites due to loss of SIRT3 in WTCD versus *Sirt3* ^*−/−*^CD mice or WTCR versus *Sirt3* ^*−/−*^CR mice at 25 months of age in liver (Figure 2b). As noted in the major acetylation trends displayed in Figure S2d–f, many sites showed increased acetylation in *Sirt3* ^*−/−*^ mice and an additive effect in *Sirt3* ^*−/−*^CR. These sites likely reflect direct SIRT3 targets that become hyper‐acetylated under CR. Surprisingly, we identified sites which were hypoacetylated in *Sirt3* ^*−/−*^ mice versus WT counterparts under both CD and CR (*Sirt3* ^*−/−*^CD vs. WTCD: 65 sites, *Sirt3* ^*−/−*^CR vs. WTCR: 31 sites), and sites that were hypoacetylated in *Sirt3* ^*−/−*^CR versus WTCR mice but not *Sirt3* ^*−/−*^CD versus WTCD mice (11 sites) (Table S2b). These sites show opposite behaviors to those expected, but yet their changes in acetylation follow a SIRT3‐dependent manner. This is especially interesting for sites that are hypoacetylated only in CR when Sirt3 is absent. These observations reveal a set of acetylation sites that are dependent on CR and SIRT3, but would not be direct substrate targets of SIRT3. The acetyl proteomics indicate major changes to the acetylation status of mitochondrial proteins dependent on age, genotype, and diet, and that the unique set of expected and unexpected changes under both CR and loss of SIRT3 highlights the molecular pathways in *Sirt3* ^*−/−*^CR mice associated with increased lifespan.

To identify the mitochondrial processes that are likely perturbed due to *Sirt3* ^*−/−*^ status and CR at old age, we performed functional cluster analysis of KEGG pathways (Figure 2c, DAVID 6.8) (Dennis et al., 2003). We compared the KEGG pathway enrichment analysis and found many similarities between the enriched pathways in the CD‐fed *Sirt3* ^*−/−*^ vs WT mice and the CR‐fed *Sirt3* ^*−/−*^ vs WT mice comparisons. In both comparisons (Figure 2c) significant enrichment of major metabolic pathways was apparent, including TCA cycle, valine, isoleucine and leucine degradation, and fatty acid degradation. In the most affected pathways (Figure 2d), 52 acetylation sites (>5% stoichiometry change, *p* < 0.1) were identified in fatty acid oxidation, TCA cycle, electron transport chain (ETC), and branched‐chain amino acids (BCAA) metabolism. Among these sites, 23 sites were previously noted in Hebert et al. (2013) and 29 sites are newly identified. In addition to increased acetylated sites due to loss of SIRT3, we observed 44% of sites displaying lower acetylation stoichiometry in *Sirt3* ^*−/−*^CD and CR mice relative to their WT counterparts, again suggesting that alterations in acetylation occurred in unexpected combinations of sites that decreased or increased in acetylation. Regardless of the direction change in acetylation, the cluster analysis suggests a functional alteration in oxidative metabolism is likely associated with the extended lifespan observed in *Sirt3* ^*−/−*^CR mice (Figure 2c,d).

### 2.3 Loss of SIRT3 limits aerobic fitness due to reduced mitochondrial respiration capacity

**FIGURE 3:**
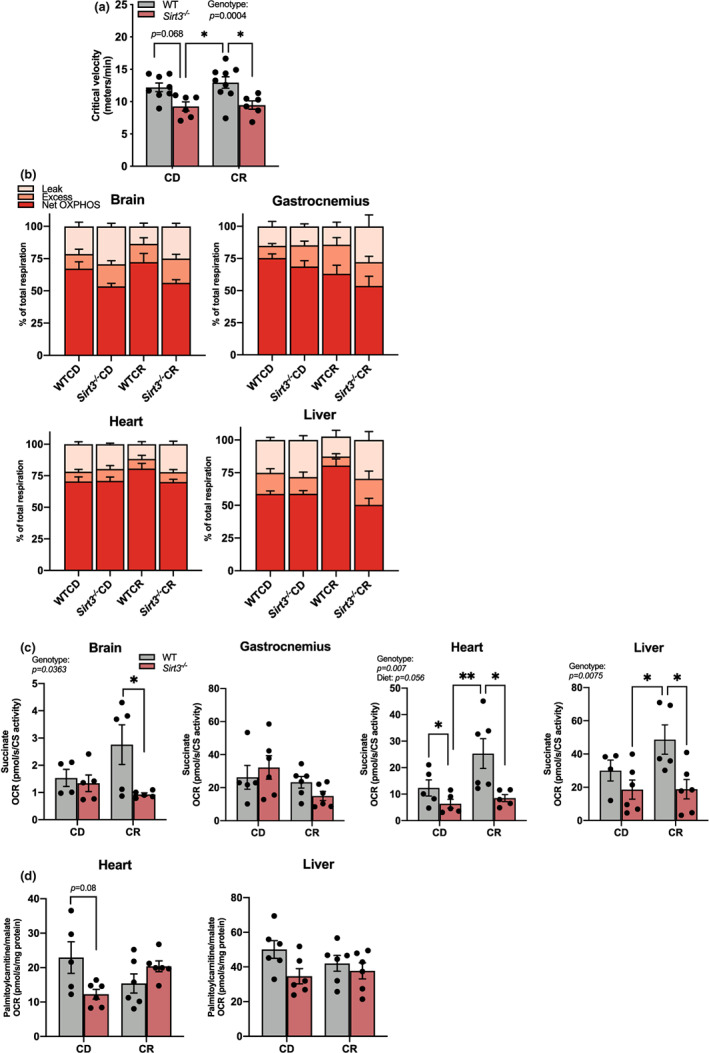
Loss of SIRT3 limits aerobic fitness due to reduced mitochondrial respiration capacity. (a) The critical velocity of 25‐month‐old WTCD, Sirt3^−/−^CD, WTCR, Sirt3^−/−^CR mice, n = 6–8 per group. (b) Percentages of leak, excess, and net OXPHOS respiration to total respiration in permeabilized brain, gastrocnemius, heart and liver from 24 h fasted 25‐months‐old mice, n = 5–6 per group. Each of these respiration parameters was assessed by oxygen consumption rate (OCR). Total respiration (electron transfer capacity) was assayed using pyruvate, glutamate, malate, succinate as substrates upon mitochondrial uncoupler FCCP addition. Leak respiration was assayed using pyruvate, glutamate, malate in the absence of ADP. Coupled respiration was assayed using pyruvate, glutamate, malate, succinate in the presence of ADP. Net‐OXPHOS respiration was calculated by subtracting leak respiration from coupled respiration. Excess respiration is calculated by subtracting coupled respiration from electron transfer capacity. Percentages of leak, excess, and OXPHOS respiration to total respiration were calculated by (OCR of each respiration parameter)/(ORC of total respiration) × 100%. See Figure S3a for OCR of each respiration parameter and Figure S3b for statistical details. (c) Succinate‐dependent respiration capacity was assessed using succinate as substrate in the presence of FCCP and rotenone, n = 4–6 per group. (d) FAO‐dependent coupled respiration was assayed in liver and heart from 24 h fasted 25‐month‐old mice using palmitoylcarnitine, malate as substrates in the presence of ADP, n = 5–6 per group. Data were analyzed by two‐way ANOVA followed by multiple t tests. *p* value reported for each comparison is corrected by Tukey's test. Results are plotted as mean ± SEM. *p ≤ 0.05; **p ≤ 0.01. Significant (*p* ≤ 0.05) diet effect, genotype effect, and/or diet and genotype interaction for each experiment are indicated in figures. ANOVA, analysis of variance; CD, control diet; CR, calorie restriction; FAO, fatty acid oxidation; SEM, standard error of the mean; SIRT3, sirtuin3

To understand how altered acetylation of pathways identified in the acetyl‐proteomics might affect mitochondrial oxidative metabolism in aged *Sirt3* ^*−/−*^CR mice, we first assessed whole‐animal bioenergetics through aerobic exercise capacity. At 25 months of age, WTCD, *Sirt3* ^*−/−*^CD, WTCR, *Sirt3* ^*−/−*^CR mice were subjected to four sessions of treadmill exercise at different velocities until exhaustion, and the critical velocity, a measure of aerobic exercise capacity, was obtained (Scariot et al., 2019). Both *Sirt3* ^*−/−*^ groups performed poorly, displaying critical velocity for *Sirt3* ^*−/−*^CD and *Sirt3* ^*−/−*^CR mice that were 24% and 27% slower than that of their diet matched control animals (Figure 3a).

Next, we investigated whether reduced aerobic fitness in *Sirt3* ^*−/−*^ mice can be explained by compromised mitochondrial respiration given that subunits of ETC complexes were one of the most prominent SIRT3 target groups and found to be inhibited under hyperacetylation induced by SIRT3 ablation (Ahn et al., 2008; Bao et al., 2010; Horton et al., 2016; Parodi‐Rullán et al., 2018) (Table S2). To this end, we measured ex‐vivo mitochondrial respiration in permeabilized brain, gastrocnemius, heart, and liver from 25‐month‐old treatment groups. Using high resolution respirometry with pyruvate, glutamate, malate, and succinate as substrates, we found that WTCR mice exhibited the highest electron transport capacity in heart (Figure S3a) and the highest percentage of net OXPHOS respiration relative to total respiration in heart and liver (Figure 3b, see Figure S3b for statistics) among four treatment groups, consistent with previous reports that CR preserves respiration efficiency in aged mice (Lanza et al., 2012; Weindruch et al., 1980). In the CD group, net OXPHOS respiration (Figure S3a) and its percentage to total respiration (Figure 3b) are largely comparable in gastrocnemius muscle, heart, and liver between genotypes, with a ~20% reduction (*p* = 0.042) in percentage of net OXPHOS respiration found in brain mitochondria from *Sirt3* ^*−/−*^ relative to WT mice. In contrast, under CR, we observed pronounced reductions in net OXPHOS respiration (Figure 3b) and its percentage relative to total respiration capacity (Figure S3a) in brain, heart and liver in *Sirt3* ^*−/−*^CR compared to WTCR mice. These respiration phenotypes suggest that both SIRT3 and CR can play a role in preserving mitochondrial respiration capacity and bioenergetics in old age and that reduced net OXPHOS respiration is a consistent feature of *Sirt3* ^*−/−*^CR mice.

To pinpoint the affected respiration pathway(s), we parsed out substrate‐dependent respiration and conducted enzymatic activity assays for the 25‐month‐old treatment groups. No significant difference in NADH‐linked substrates (pyruvate, glutamate, and malate) coupled respiration between genotypes nor between diets was observed (Figure S3c). Succinate‐dependent respiration capacity, however, was significantly reduced in *Sirt3* ^*−/−*^CR liver and heart relative to WTCR (Figure S3c). In CD‐treated groups, succinate‐dependent respiration capacity showed minor difference in heart between WT and *Sirt3* ^*−/−*^. Enzymatic activity assays revealed that SIRT3 and CR indeed had the more prominent impact on Complex II activity compared to that of Complex I (Figure S3d,e). Moreover, we determined if fatty acid oxidation (FAO)‐dependent respiration was limited in permeabilized liver and heart from *Sirt3* ^*−/−*^ animals using palmitoylcarnitine and malate as substrates. Interestingly, palmitoylcarnitine‐dependent coupled respiration (Figure 3d) and its net OXPHOS control efficiency (Figure S3f) was similar between WTCR and *Sirt3* ^*−/−*^CR mice in both liver and heart. In contrast to mice on the CD diet, palmitoylcarnitine‐dependent OXPHOS control efficiency (Figure S3f) was significantly lower in *Sirt3* ^*−/−*^ mouse heart relative to WT. These results suggest that the reduced Complex II respiration in SIRT3‐deficient mice provides a molecular basis for lowered whole‐body aerobic exercise capacity. But notably, CR treatment allows *Sirt3* ^*−/−*^ mice to maintain FAO‐dependent respiration.

### 2.4 *Sirt3* ^*−/−*^ mice under CR display trends in metabolic parameters that are consistent with faster switching from glucose to fatty acid oxidation during the postprandial period

**FIGURE 4:**
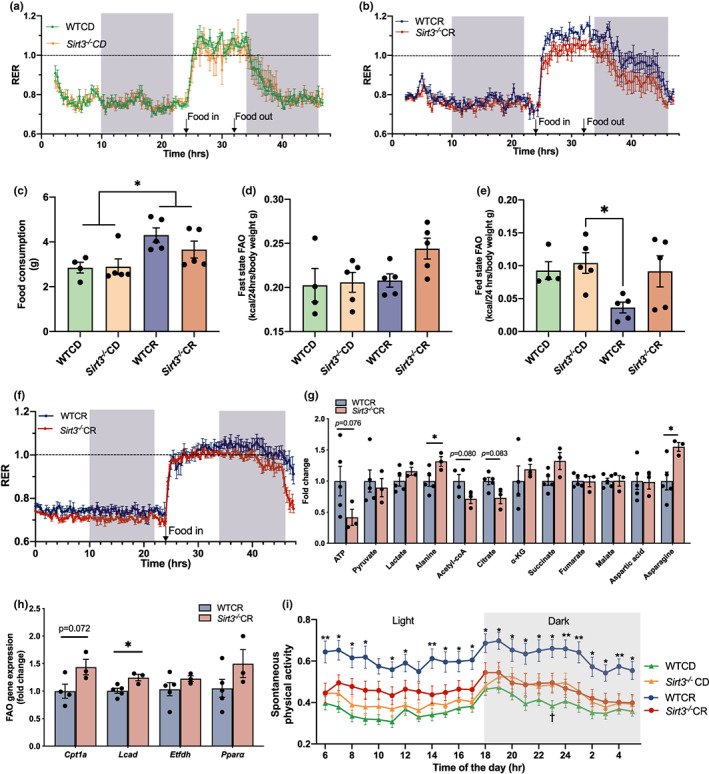
Sirt3^−/−^ mice under CR display faster switching from glucose to fatty acid oxidation during the postprandial period. (a–c) Respiratory exchange ratio (RER) and food consumption of 25‐month‐old WTCD, Sirt3^−/−^CD, WTCR, Sirt3^−/−^CR mice in metabolic chamber experiments, n = 5. Duration of food availability is indicated in both figures, which is the interval between food in and food out. Results are plotted as mean ± SEM. See Figure S4b,c for RER statistics. (d, e) FAO during fast and fed states for mice is shown in (a, b). Data reported here are corrected to body weight. (f) RER of 25‐month‐old WTCR, Sirt3^−/−^CR mice, n = 5,3. Food was provided at the 24th hour of the experiment, indicated as food in. The normal daily amount of food was provided, and no food was taken out during the experiment. See Figure S4h,i for RER statistics. (g) Fold change of major TCA metabolites in 25‐month‐old CR‐treated mice liver after a 6‐h refeeding period, n = 5 (WTCR), 3 (Sirt3^−/−^CR). (h) Fatty acid oxidation gene expression in 25‐month‐old CR‐treated mice liver after a 6‐h refeeding period. Gene expressions are normalized to β‐2‐microglobulin and relative to WTCR values, n = 5 (WTCR), 3 (Sirt3^−/−^CR). (c–e) were analyzed by two‐way ANOVA followed by multiple t tests. *p* value reported for each comparison is corrected for multiple comparisons using the Tukey's test. (g, h) were analyzed by Welch's t tests. Results are plotted as mean ± SEM. *p ≤ 0.05; **p ≤ 0.01 (i) Spontaneous physical activity in arbitrary units at the light and dark phases for 25 months WTCD, Sirt3^−/−^CD, WTCR, Sirt3^−/−^CR mice over a 19‐day period, n = 6 per group. Each data point represents the 19‐day average of spontaneous activity of all mice in the same treatment group at the indicated hour of the day. *Represents the statistical significance between WTCR versus Sirt3^−/−^CR (*p ≤ 0.05; **p ≤ 0.01); ^†^Represents the statistical significance between WTCD versus Sirt3^−/−^CD (^†^ *p* ≤ 0.05). Both comparisons were analyzed by multiple t tests. *p* reported for each time point is corrected for multiple comparison. ANOVA, analysis of variance; CD, control diet; CR, calorie restriction; FAO, fatty acid oxidation; SEM, standard error of the mean; SIRT3, sirtuin3

In light of the critical velocity and substrate‐dependent respiration phenotypes obtained from the four treatment groups, we speculated that *Sirt3* ^*−/−*^CR mice display unique metabolic features. The four groups of 25‐month‐old mice were subjected to a metabolic chamber experiment that consisted of a 24‐h fasting period followed by a refeeding period with 8 h of food provision (Figure 4a,b; Figure S4b,c). Diet‐matched groups consumed a similar amount of food during the 8‐hour food provision period (Figure 4c). During the fasting period, the respiratory exchange ratio (RER) was not significantly different among the treatment groups, with RERs ranging between 0.7 and 0.8, indicating that fatty acids were the preferred energy source. Upon feeding, RER increased to ≥1 for all groups, reflecting a fuel switching to the predominant use of carbohydrate as an energy source, accompanied by fatty acid synthesis before returning to FAO in the fasted state (Bruss et al., 2010; Mitchell et al., 2019). We observed a trend of smaller amplitudes of RER elevation upon feeding in *Sirt3* ^*−/−*^CR mice and a shorter duration of the RER ≥1 period as compared to that of WTCR mice, whereas the comparison of WTCD and *Sirt3* ^*−/−*^CD mice showed no such trend. Notably, the shape of the RER curve for CR mice was different from that of CD mice. Instead of a RER drop to baseline (fasted state RER), CR mice maintained a RER ~0.8–0.9 for 6 h, suggesting a longer period of mixed fuel utilization compared to CD mice. We noted that CR mice consumed significantly more food in the feeding period (Figure 4c), which may have contributed to this observation. We also noted that food consumption of KOCR mice was trending lower, which may play a role in the earlier reduction in RER compared to WTCR. Energy expenditure under both fast and fed states was calculated (Figure S4d,e), along with analysis of volume of O_2_ consumption and CO_2_ generation (Figure S4f,g). From RER and energy expenditure measurements, fed and fasted state FAO were calculated (Figure 4d,e). We observed a trend of increased FAO in *Sirt3* ^*−/−*^CR mice compared to WTCR mice. To control for the confounding impact of weight on energy expenditure, we measured the impact of genotype (WT vs. *Sirt3* ^*−/−*^) and diet (CD vs. CR) on energy expenditure using analysis of covariance (ANCOVA) with body weight as a covariate. We split mice into fed/fasted and dark/light groups, and performed ANCOVAs on each set. For fed mice at both dark and light times of day, energy expenditure was not significantly affected by diet or genotype (Table S4). For mice fasted during the dark period, this was also true, although body weight did have a significant impact on energy expenditure, with higher body weights resulting in higher energy expenditures. For fasted mice in the light phase, while controlling for body weight, energy expenditure was significantly different between genotypes, with energy expenditure significantly increased in the KO group, although there was no effect of diet.

Given the every‐other‐day feeding protocol in the current lifespan experiment for both CD and CR animals (Pugh et al., 1999), CR animals generally consumed their daily allotment of food within 12–16 h without altered eating behavior observed between genotypes. To capture daily RER fluctuations and estimate the duration of the predominant carbohydrate utilization period in CR mice, we repeated the metabolic chamber experiments using a different set of CR animals with 24 h of fasting followed by a refeeding period in which the normal daily food allotment used in the lifespan study was provided, and no food was removed during the experiment. These conditions mimic the normal food intake routine of this cohort of animals throughout their lifespan. Again, a trend of smaller amplitude of RER in response to food intake and a shorter duration of RER ≥1 were observed in *Sirt3* ^*−/−*^CR relative to WTCR mice (Figure 4f; Figure S4h,i), consistent with the RER pattern observed in Figure 4a,b. Energy expenditure (Figure S4j,k), O_2_ consumption, and CO_2_ generation (Figure S4l,m) were comparable between WTCR and *Sirt3* ^*−/−*^CR mice, and fasted state FAO trended higher in *Sirt3* ^*−/−*^CR mice (Figure S4n). Our observations suggest that *Sirt3* ^*−/−*^CR mice are able to switch to carbohydrate as the predominant fuel source upon feeding, but may be less capable of maintaining carbohydrate utilization during the fed state. Possibly, *Sirt3* ^*−/−*^CR animals experience an earlier switch to FAO‐dependent metabolism (fasting state) compared to WTCR.

To further investigate whether *Sirt3* ^*−/−*^CR mice have compromised glucose oxidation during the fed state, we performed liquid chromatography–mass spectrometry‐based metabolite profiling in liver, heart, and muscle of 6‐h refed mice. Compared to WTCR mice, the *Sirt3* ^*−/−*^CR group exhibited a trend of reduced levels of ATP (*p* = 0.07), acetyl‐CoA (*p* = 0.08), and citrate (*p* = 0.08) accompanied with a significant accumulation of alanine and asparagine in liver (Figure 4g). We also observed a significant reduction of citrate and a trend of accumulation of aspartate (*p* = 0.06) and asparagine (*p* = 0.06) in the heart (Figure S4p). Reduced pyruvate and alpha ketoglutarate were observed in the heart of *Sirt3* ^*−/−*^CD mice compared to WTCD mice (Figure S4r), and no difference was detected in liver (Figure S4q). No difference in TCA metabolites was found in gastrocnemius among all groups except increased pyruvate noted in CR groups (Figure S4s). Next, we examined whether FAO genes were upregulated at an earlier time upon feeding, which could explain the decline of the RER in *Sirt3* ^*−/−*^CR mice. We measured transcript levels of key FAO genes, including *Cpt1α*, *Lcad*, *Etfdh*, and *Pparα* in 6‐h refed liver from 25‐month‐old WTCR and *Sirt3* ^*−/−*^CR mice (Figure 4h). After 6 h of food provision, *Lcad* transcripts were significantly increased in *Sirt3* ^*−/−*^CR mice and a trend for Cpt1α increase was observed (*p* = 0.07), consistent with the *Sirt3* ^*−/−*^‐ and CR‐dependent fuel utilization adaptation observed in whole body RER studies.

Next, we examined pontaneous physical activity (SPA) as an important component of daily energy expenditure (Manini, 2010) and as a parameter linked to healthspan and lifespan (Garcia‐Valles et al., 2013; Lieberman et al., 2021). Here, we observed that loss of SIRT3 blunted CR‐mediated higher SPA in a 19‐day recording period (Figure 4i). These results indicate that SIRT3 is required for higher SPA under CR. The net consequence of SIRT3 loss under CR is a longer‐lived animal that is less active, has reduced exercise and oxidative capacity, but may display increased fat utilization as an energy source.

## 3 DISCUSSION

Caloric restriction is a widely studied regimen in mice that robustly extends both healthspan and lifespan (Fontana & Partridge, 2015; Mitchell et al., 2019; Weindruch et al., 1986; Zhang et al., 2013). Previous work revealed that CR can stimulate SIRT3 expression (Palacios et al., 2009; Schwer et al., 2009), and through deacetylation of mitochondrial enzymes enhances metabolic flux of pathways often dysregulated in aging and age‐related disorders (Ansari et al., 2017; Kincaid & Bossy‐Wetzel, 2013; McDonnell et al., 2015). Here, we set out to investigate the importance of SIRT3 in CR‐mediated longevity. We report that SIRT3 is dispensable for CR‐dependent longevity and unexpectedly find that SIRT3 ablation further extends maximum lifespan under CR. Maximum lifespan is a parameter thought to correlate better with rates of aging as compared to average lifespan, since it is less likely to be influenced by strain‐specific diseases that can shorten life (Weindruch & Sohal, 1997). Though it is unclear if the extended maximum lifespan of *Sirt3* ^*−/−*^CR mice is due to a retardation of the aging process, lifelong metabolic adaptations in *Sirt3* ^*−/−*^CR mice may confer stress resistance late in life. SIRT3 deficiency attenuates succinate‐dependent respiration but preserves palmitate‐dependent respiration in heart and liver of aged CR‐treated mice. Consistent with the ex vivo respiration analyses, *Sirt3* ^*−/−*^CR mice were less able to maintain carbohydrate‐derived energy production and displayed faster fuel switching to FAO during the postprandial period, leading to a longer fasting state relative to WTCR animals. We note that our results derived from metabolic cage experiments were limited by the number of mice available at the end of this study, but collectively with other analyses are consistent with a fuel‐switching difference between *Sirt3* ^*−/−*^CR versus WTCR groups. Also, we acknowledge that only male mice were utilized in this study and that sex‐specific phenotypic differences are often observed in such studies. Despite longer maximum lifespan, *Sirt3* ^*−/−*^CR mice display altered fuel utilization, reduced exercise capacity, and reduced spontaneous activity.

Managing ROS production and detoxification has been proposed as a major SIRT3 function (Bause & Haigis, 2013; Liu et al., 2017; Zeng et al., 2014). In the current study, we found no compelling evidence that Sirt3 deletion leads to ROS‐dependent dysfunction. It is important to note that most prior aging and SIRT3 studies used C57BL/6J mice that lack functional NNT, a major contributor to NADPH production. The present study was conducted using the C57BL/6NJ × C57BL/6J *Nnt* ^*+/+*^ genetic background. Likely, loss of both SIRT3 and NNT in previous studies generated a more severe mitochondrial phenotype in which levels of NADPH and reduced glutathione (GSH) were significantly reduced (Qiu et al., 2010; Someya et al., 2010). We found that in the presence of functional NNT, NADPH and GSH levels were maintained in all treatment groups, strongly suggesting that the aging and metabolic phenotypes in the current study are independent of oxidative stress. Another important difference in our experimental design is the choice of a CD that provides a fixed amount of 16% less calories as compared to ad libitum food intake. This CD minimizes obesity‐induced complications and allows a better comparison of the health benefits of CR relative to a non‐pathological diet (Pugh et al., 1999).

Loss of Sirt3 leads to a composite set of acetylation trends on mitochondrial proteins. While CR, age, and loss of Sirt3 combined led to more sites of hyperacetylation, a detailed clustering analysis revealed sub‐groups of sites in metabolic proteins that were dependent on both CR and Sirt3, but displayed decreased acetylation in *Sirt3* ^*−/−*^CR mice. Fasting and CR increase protein acetylation in a tissue‐dependent manner (Dittenhafer‐Reed et al., 2015; Schwer et al., 2009), likely by promoting FAO that increases mitochondrial acetyl‐CoA production and consequently protein acetylation (Mezhnina et al., 2020; Pougovkina et al., 2014). Aging was also associated with elevated mitochondrial acetylation in various tissues (Joseph et al., 2012; Zeng et al., 2014). In current study, CR, aging, and SIRT3 ablation increase overall liver mitochondrial acetylation. However, detailed analysis of our acetyl‐proteomics suggests that each variable contributes to acetylation sites to different extents and that these lysine sites can be independent or summations of the variables. Compared to CR and aging, loss of SIRT3 yielded the fewest number of lysine sites that showed increased stoichiometry (Figure S2a). This result is not surprising given that CR and aging have broader impacts that affect acetyl‐CoA level, mitochondrial integrity, and metabolism in general. In addition, we employed a stoichiometry‐based MS approach that does not introduce possible bias of overestimation from use of acetyl‐antibody enrichment. SIRT3‐dependent acetylation sites are primarily enriched with proteins from oxidative metabolism pathways, and appear to counteract a rise in protein acetylation in a subset of mitochondrial proteins under metabolic stress. KEGG pathway enrichment analysis of hyperacetylated proteins in old *Sirt3* ^*−/−*^CR mice demonstrated that BCAA degradation and fatty acid degradation were highly enriched within this particular subset. Some of the BCAA pathway acetylation sites with the largest increase (Table S2) in the old *Sirt3* ^*−/−*^CR mice compared to the baseline condition, the same age WTCD mice, included ACAD8 K220 (37% increase), ACAT1 K178 (16% increase), AUH K186 (10% increase), and ALDH7A1 K462 (50% increase). These sites were of particular interest because structural analyses indicate these sites are in close proximity to active sites or dimerization interfaces. For example, ACAD8 K220, ACAD8 K178, and AUH K186 are all adjacent to nearby NAD+, FAD+, and enoyl‐CoA binding pockets, respectively (Kurimoto et al., 2001; Battaile et al., 2004, PDB2F2S). ALDH7A1 is a homotetramer, and K462 is found at the interface between the dimers of dimers. Others have suggested that NAD+ binding promotes the tetramerization of ALDH7A1 and its activation; therefore, acetylation at this interface could be an important regulator of ALDH7A1 dehydrogenase activity (Korasick et al., 2018; Luo et al., 2015). Recent studies (Richardson et al., 2021; Yu et al., 2021) have shown that manipulation of BCAA level and its metabolism may lead to a profound impact on metabolic parameters, including glucose control, adiposity, and longevity. Future studies are needed to determine the molecular and phenotypic consequence of SIRT3 loss in BCAA metabolism to resolve the influence of SIRT3 on aging mechanisms.

Unexpectedly, we identified a subgroup of acetylation sites that showed hypoacetylation in SIRT3‐deficient animals on the CR diet. Though regulated by SIRT3 under CR, these sites are unlikely to be direct deacetylation substrates of SIRT3. The acetylation status of these sites could reflect the metabolic adaptation due to loss of SIRT3, leading to the unique phenotypes observed in *Sirt3* ^*−/−*^CR mice. It is intriguing to speculate that acetylation of these sites is linked to acetyl‐CoA availability, which could be positively regulated by SIRT3, for example, through PDH (Jing et al., 2013). Loss of SIRT3 leads to downregulation of Pyruvate Dehydrogenase (PDH) activity and reduced protein acetylation of these acetyl‐CoA‐sensitive sites, which include the site K304 on ACAT1 that was one of the most reactive lysine residues during non‐enzymatic acetylation with acetyl‐CoA (Baeza et al., 2016). Consistent with this idea, *Sirt3* ^*−/−*^CR compared to WTCR mice show decreased levels of acetyl‐CoA and citrate. Among this group of hypoacetylated sites in the *Sirt3* ^*−/−*^CR condition, additional FAO pathway proteins include HADHA, ACAA2, and ECI1. Such protein acetylation sites may serve as an acetyl‐CoA sensor and contribute to the FAO metabolic adaptation observed in *Sirt3* ^*−/−*^CR mice.

Manipulation of SIRT3 expression results in clear changes in respiration phenotypes, with significant differences observed between the CR‐treated groups. SIRT3 ablation blunted CR‐mediated respiration preservation and limited net OXPHOS respiration in *Sirt3* ^*−/−*^CR animals, suggesting that both CR and SIRT3 are needed to maintain maximal bioenergetic capacity in aged mice. Notably, this CR and SIRT3‐dependent respiratory phenotype is tissue‐dependent. Liver and heart displayed additive effects, whereas the brain showed only a genotype effect, and muscle showed neither genotype nor diet‐induced effects. We also identified Complex II as the major contributor of reduced respiration in *Sirt3* ^*−/−*^CR mice, consistent with studies (Cimen et al., 2010; Finley et al., 2011; Horton et al., 2016) showing that SIRT3 directly targets Complex II and restores activity through deacetylation. Several studies have also reported reduced Complex I activity in response to SIRT3 deletion (Ahn et al., 2008; Lantier et al., 2015; Williams et al., 2019). Although Complex I displayed altered function, compared to Complex II, Complex I activity and NADH‐linked respiration were less affected in all tissues tested, suggesting Complex II‐dependent respiration is more prone to dysfunction under current experimental settings. Intriguingly, despite a significant reduction in respiration (heart and liver) from *Sirt3* ^*−/−*^CR animals using carbohydrate‐derived substrates (pyruvate, malate, succinate), these mice maintained comparable palmitoylcarnitine, malate‐dependent coupled respiration, indicating FAO is not limited. Recent studies (Lantier et al., 2015; Williams et al., 2019) report an enhanced palmitoylcarnitine/malate‐dependent respiration phenotype in muscle of high‐fat diet‐fed *Sirt3* ^*−/−*^ mice. While these are dramatically different dietary models (high‐fat diet vs. CR), the findings demonstrate that *Sirt3* ^*−/−*^ mice can preserve FAO capacity under both diets despite altered mitochondrial acetylation. Together, maintained/enhanced FAO respiration under CR or a high‐fat diet, and the composite trends in acetylation challenge the generalized idea that mitochondrial hyperacetylation limits FAO (Hallows et al., 2011; Hirschey et al., 2010; Tsuda et al., 2018) and instead highlights the importance of the dietary regime in the context of SIRT3‐dependent mitochondrial regulation.

*Sirt3* ^*−/−*^CR mice displayed faster fuel switching from glucose to FA compared to WTCR animals during the postprandial period. This was evident in RER and amount of FAO (Figure 4a–f) showing a trend of increased FAO during the postprandial period in *Sirt3* ^*−/−*^CR relative to WTCR mice. This may be in part due to limited Complex II function and partial redirected carbon flux to alanine and asparagine, indicating reduced glucose oxidation‐dependent energy production. Unlike glucose oxidation, FAO requires an extra ETC component, the flavoprotein‐ubiquinone oxidoreductase (ETF‐QO, or ETFDH), to oxidize FADH_2_ harvested from beta‐oxidation (Gnaiger & MitoEagle Task Group, 2020; Goetzman, 2011). During acyl‐CoA chain‐shortening cycles, NADH and FADH_2_ are produced and further oxidized by Complex I and ETFDH, respectively. Switching to FAO may lead to more ETFDH‐dependent electron transfer rather than transfer through Complex II. A recent study (Kľučková et al., 2020) reported that SDH‐deficient murine chromaffin cells could maintain efficient FAO respiration that are higher than wildtype cells when palmitoylcarnitine is provided. Consistent with these observations, HepG2 cells treated with SDH inhibitor 3‐NPA blocks succinate‐dependent respiration completely but affects fatty acid oxidation minimally (Y. Qin, J. M. Denu, unpublished observation). With only weakly affected Complex I observed in our study, FAO could be preserved in *Sirt3* ^*−/−*^CR mice by increased flux through ETFDH, serving as a compensatory mechanism to drive FAO‐dependent energy generation. Future studies are needed to determine whether compromised Complex II plays a role in fuel switching.

The early shift to FAO may lead to a longer overall FAO time and ultimately a longer fasting time in *Sirt3* ^*−/−*^CR compared to WTCR mice. An increased fasting period has been a feature of meal‐fed CR models, in which CR animals quickly consume their food allotment and fast until the next meal (Acosta‐Rodríguez et al., 2017). Intriguingly, Pak et al. (2021) found that even without CR, increasing fasting time alone can promote CR‐like metabolic phenotypes, highlighting the importance of fasting time towards CR benefits. Both alternate day feeding and daily fasting increase longevity in mice, and the beneficial effects of these interventions have been shown to be independent of caloric intake (Mattson et al., 2014; Mitchell et al., 2019). Fasting is also associated with improved intestinal stem cell function in aging (Mihaylova et al., 2018). Interestingly, the long‐lived Ames dwarf and GHR‐knockout mice display increased reliance on fatty acid oxidation as a fuel source (Bartke & Westbrook, 2012). Thus, existing studies have linked increased fasting or increased fatty acid oxidation with health benefits in rodents. We speculate that the additional fatty acid oxidation dominant period may play a role in extending the maximum lifespan of *Sirt3* ^*−/−*^CR mice. Also, the ability to maintain similar fat content among the *Sirt3* ^*−/−*^CR and WTCR groups might be explained by the lower SPA in *Sirt3* ^*−/−*^CR which could spare fat mass consumption over their lifetime. While the difference in SPA is significant at 24.5%, this difference would be too small to capture in the energy expenditure (EE) estimates from the metabolic cage experiments. Estimates from literature put SPA as only 5%–20% of total EE. The feeding protocol used in the current study involves an every‐other‐day feeding schedule for both CD and CR animals. This design increases fasting time between feedings and provides a more rigorous examination on the effects of a CR regime, as compared to studies that use ad libitum feeding for controls. Despite increased lifespan, *Sirt3* ^*−/−*^CR mice are less aerobically fit compared to WTCR mice due to their inability to perform full glucose utilization (Seiler et al., 2015), emphasizing the role of SIRT3 in preserving aerobic capacity.

Taken together, our findings reveal that SIRT3 is required for optimal aerobic fitness during aging but not for CR‐mediated longevity. The lack of observable skeletal muscle differences in molecular analyses suggests that other tissues (i.e., brain, heart and/or liver), which displayed clear effects with SIRT3 loss, are more likely to be responsible for the whole‐animal phenotypic differences. These results highlight the uncoupling of lifespan and healthspan parameters such as aerobic fitness and SPA and address the need to comprehensively assess the factors that contribute to CR‐dependent phenotypes. Possibly, some CR‐induced factors contribute to lifespan extension, while having no impact on healthspan.

## AUTHOR CONTRIBUTIONS

**John M. Denu** and **Tomas A. Prolla:** Conceptualization. **Rashpal S. Dhillon, Yiming (Amy) Qin, Paul R. van Ginkel, Vivian X. Fu, Alexis J. Lawton, Cara L. Green, Fúlvia B. Manchado‐Gobatto**, and **Claudio A. Gobatto:** Methodology, investigation, and data collection. **Yiming (Amy) Qin**, **Rashpal S. Dhillon**, **Alexis J. Lawton**, **Cara L. Green**, **Fúlvia B. Manchado‐Gobatto**, and **Claudio A. Gobatto:** Data analysis and figure generation. **Yiming (Amy) Qin**, **John M. Denu**, **Tomas A. Prolla**, **Rashpal S. Dhillon**, and **Alexis J. Lawton**: Writing‐Original Draft. **Yiming (Amy) Qin**, **John M. Denu**, **Tomas A. Prolla**, and **Cara L. Green:** Writing‐Review & Editing. **John M. Denu**, **Tomas A. Prolla**, **Dudley W. Lamming**, **Cara L. Green**, **Fúlvia B. Manchado‐Gobatto**, and **Claudio A. Gobatto:** Resources. Vivian X. Fu and **James M. Vann:** Animal breeding and care. **John M. Denu**, **Tomas A. Prolla**, **Dudley W. Lamming**, **Fúlvia B. Manchado‐Gobatto**, and **Claudio A. Gobatto:** Funding acquisition.

## CONFLICT OF INTEREST

J.M.D. is a consultant for Evrys Bio and co‐founder of Galilei BioSciences. T.A.P. is a co‐founder of Lifegen Technologies and a scientific advisory board member of Nu Skin International Inc. and CyteGen Corporation. D.W.L. has received funding from and is a scientific advisory board member of Aeovian Pharmaceuticals, which seeks to develop novel, selective mTOR inhibitors for the treatment of various diseases. The remaining authors declare no competing interests.

## Supporting information


Figure S1
Figure S2
Figure S3
Figure S4

Appendix S1

Table S1

Table S2

Table S3
Table S4

## Data Availability

The raw data, processed data, spectral library, and the analysis logs describing the settings for the Spectronaut analyses have been deposited to the ProteomeXchange Consortium via the MassIVE partner repository with the dataset identifier MSV000087085 and PXD024961 (DOI: 10.25345/C59Z2Q). The data were processed and cleaned using an in‐house R script, which can be accessed through the GitHub link: (DOI: 10.5281/zenodo.3360892). Other data are available upon request.
