# Supplementary material for: SIRT3 deficiency decreases oxidative metabolism capacity but increases lifespan in male mice under caloric restriction
Source: Aging Cell. 2022 Oct 5;21(12):e13721. doi: 10.1111/acel.13721 (PMC9741511; doi:10.1111/acel.13721)
Supplement: Supplementary file 1 — Figure S1 Figure S2 Figure S3 Figure S4 [file ACEL-21-e13721-s003.pdf]

Fig S1.

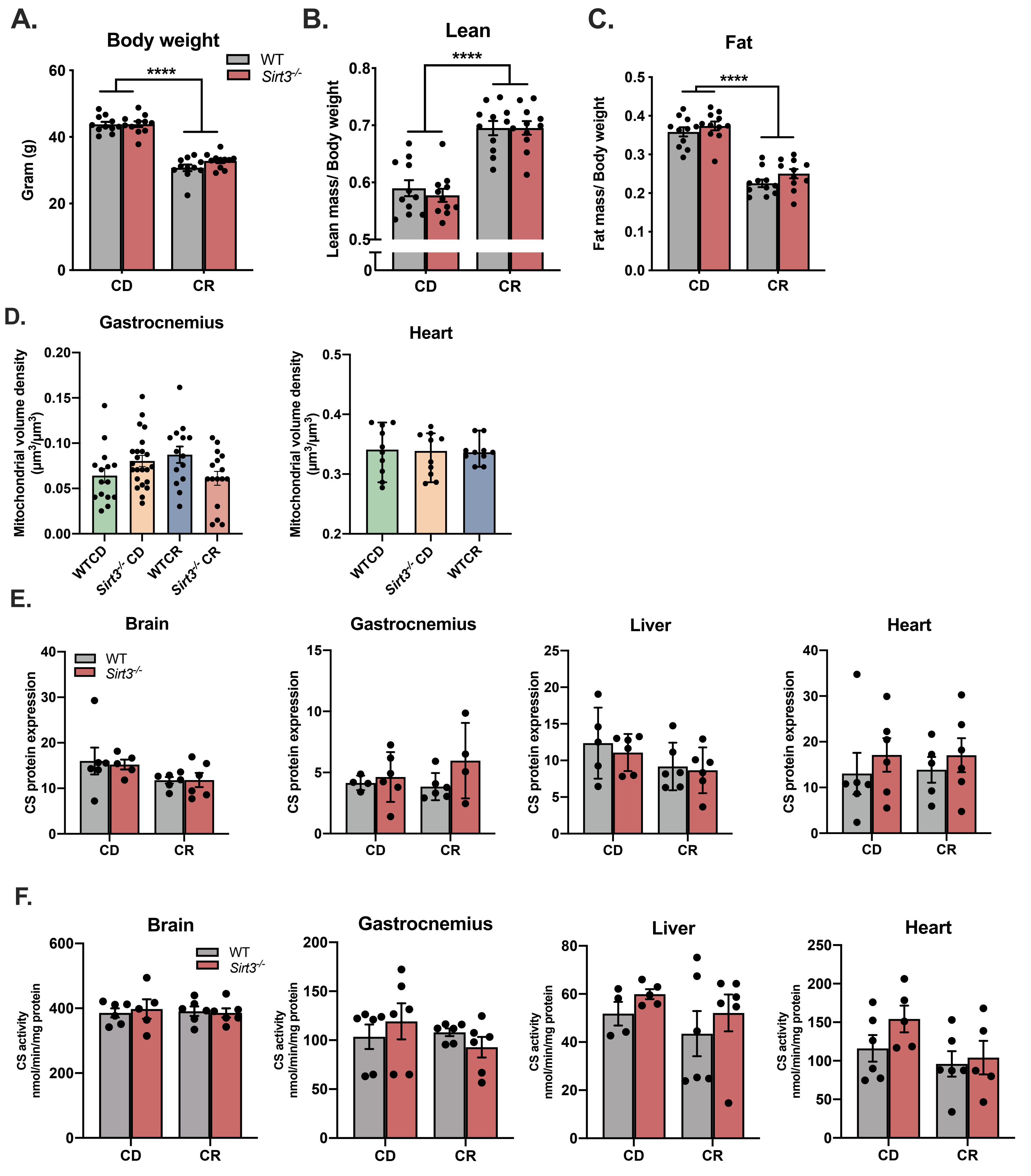
A.
Body weight
WT
Sirt3-/-
****
Gram (g)
60
40
20
0
CD
CR
B.
Lean
****
Lean mass/ Body weight
0.8
0.7
0.6
0.5
0
CD
CR
C.
Fat
****
Fat mass/ Body weight
0.5
0.4
0.3
0.2
0.1
0.0
CD
CR
D.
Gastrocnemius
Mitochondrial volume density (µm3/µm3)
0.20
0.15
0.10
0.05
0.00
WTCD
Sirt3-/- CD
WTCR
Sirt3-/- CR
Heart
0.5
0.4
0.3
0.2
WTCD
Sirt3-/- CD
WTCR
E.
Brain
Gastrocnemius
Liver
Heart
CS protein expression
WT
Sirt3-/-
CD
CR
F.
Brain
Gastrocnemius
Liver
Heart
CS activity nmol/min/mg protein
WT
Sirt3-/-
CD
CR

Figure S1.

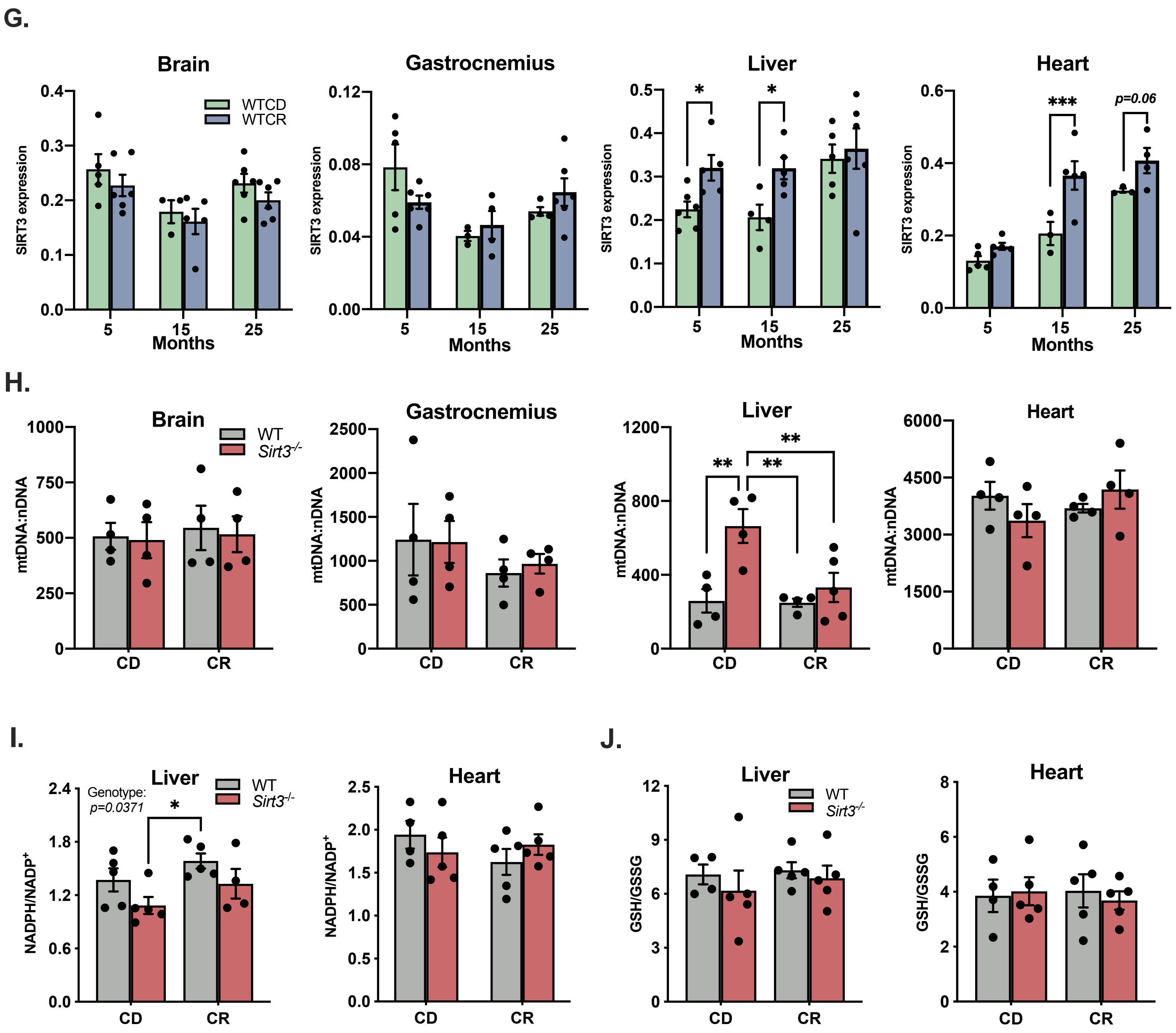

Fig S1.

A-C) Bodyweight and body composition for 25-month-old treatment groups, n=11 per group.

D) Gastrocnemius and heart mitochondrial volume density from 25-month-old mice. n=4 per groups for gastrocnemius measurement and n=3 for heart measurement. *Sirt3*$^{-/-}$CR heart mitochondrial volume density is not available in this study.

E-F) Citrate synthase expression and activity for 25-month-old treatment groups, n=5-6 per group. Citrate synthase expression was measured using automated capillary electrophoresis-based immunodetection system (WES, ProteinSimple).

G) SIRT3 protein expression for 5-, 15-, and 25-month-old WTCD and WTCR groups, n=4-6 per group. SIRT3 expression was measured using automated capillary electrophoresis-based immunodetection system (WES, ProteinSimple).

H) Ratio of mitochondrial DNA to nuclear DNA (mDNA:nDNA) for 25-month-old treatment groups, n=4 per group.

I-J) NADPH/$NADP^+$ and GSH/GSSG in liver and heart from 25-month-old treatment groups measured by LC-MS. NADP(H) and GSH(GSSG) standard curves were used for ratio calculation. n= 4-5 per group.

Data were analyzed by two-way ANOVA followed by multiple comparisons test. *p* value reported for each comparison is corrected by Tukey's test. Results are plotted as mean ± SEM. *: p≤0.05; **: p≤0.01; ***: p≤0.001.

Fig S2.

A.

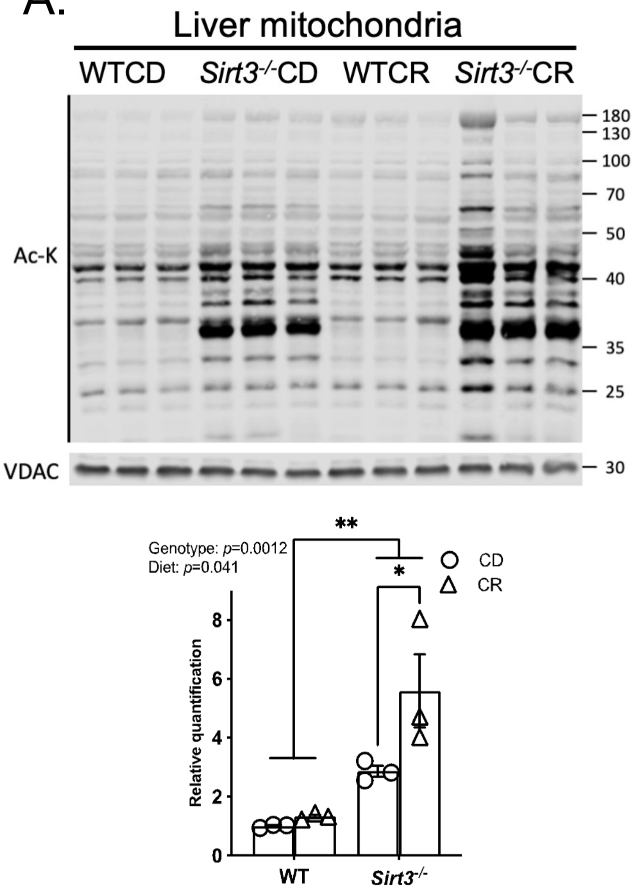

B.

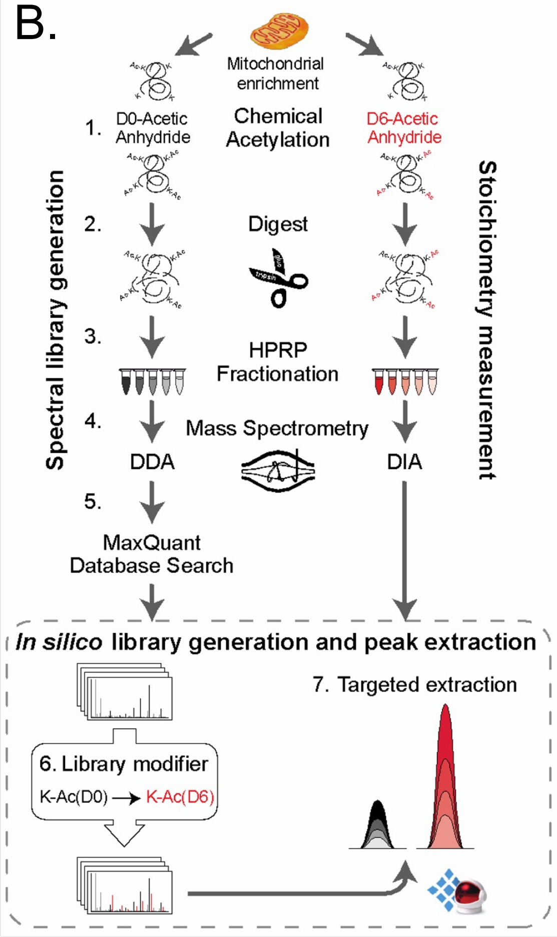

C.

Categories	CR	Age	Genotype
	Two diets (CR vs. CD), in age- and genotype-matched groups	Two ages (25 vs. 5 months), in diet- and genotype-matched groups	Two genotypes (*Sirt3*$^{-/-}$ vs. WT), in age- and diet-matched groups
Number of stoichiometry significantly changed acetyl-lysine sites (protein)	332 (205)	281 (173)	259 (162)
Number of acetyl-lysine sites showed increased stoichiometry change (protein)	185 (130)	176 (120)	139 (111)

Fig S2.

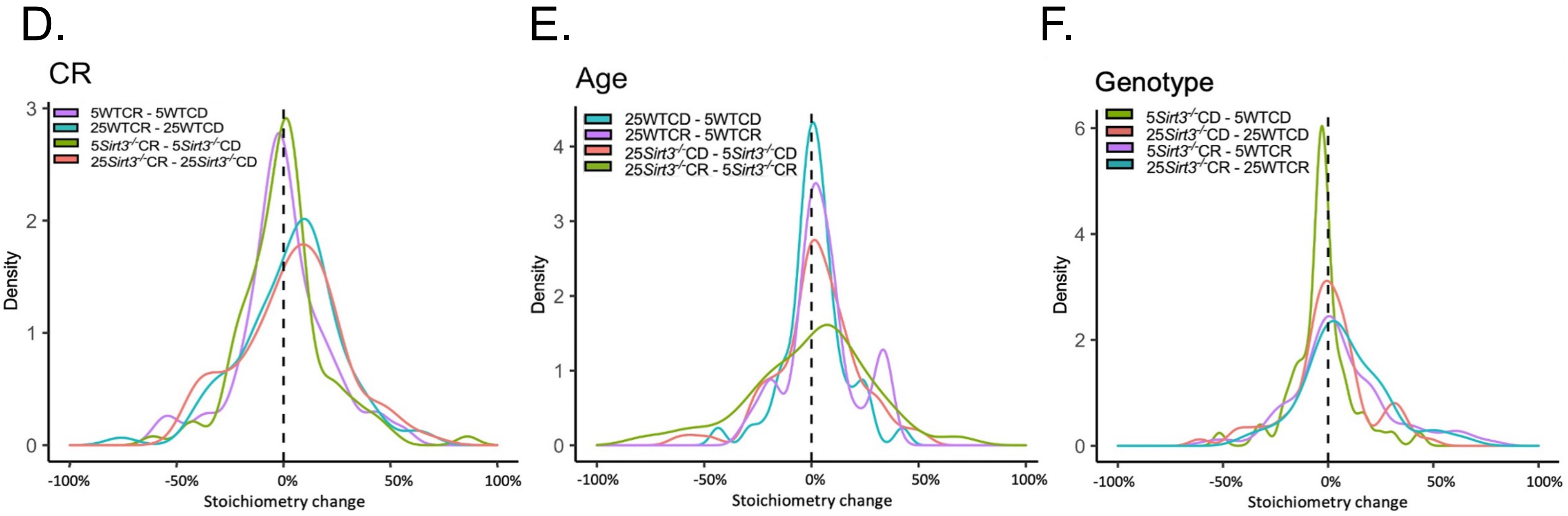

Fig S2.

A) Immunoblot of pan-acetylation, normalized to VDAC, for liver mitochondrial enrichment from 25-month-old treatment groups. n=3 per group. Data were analyzed by two-way ANOVA followed by multiple comparisons test. *p* value reported for each comparison is corrected by Tukey's test. Results are plotted as mean ± SEM. *: $p \leq 0.05$.

B) Workflow of DIA-dependent acetylation stoichiometry quantification.

C) Number of stoichiometry significantly changed ($p \leq 0.05$) acetyl-lysine sites and number of acetyl-lysine sites showed increased (>0%) stoichiometry change per factor. Number of proteins are indicated in parentheses. n=4 per group for MS analysis.

D-F) Distribution of CR-, age-, and genotype-dependent acetylation stoichiometry changes. CR-induced acetylation stoichiometry change is obtained from the stoichiometry difference between CR and CD, in age- and genotype-matched animals. Age-induced acetylation stoichiometry change is obtained from the stoichiometry difference between 25 months and 5 months, in diet- and genotype-matched animals. Genotype-induced acetylation stoichiometry change is obtained from the stoichiometry difference between Sirt3$^{-/-}$ and WT, in diet- and age-matched animals. Only significantly changed acetylation sites ($p < 0.05$) between two groups comparison are included in the density plots. n=4 per group.

Fig S3.

A.

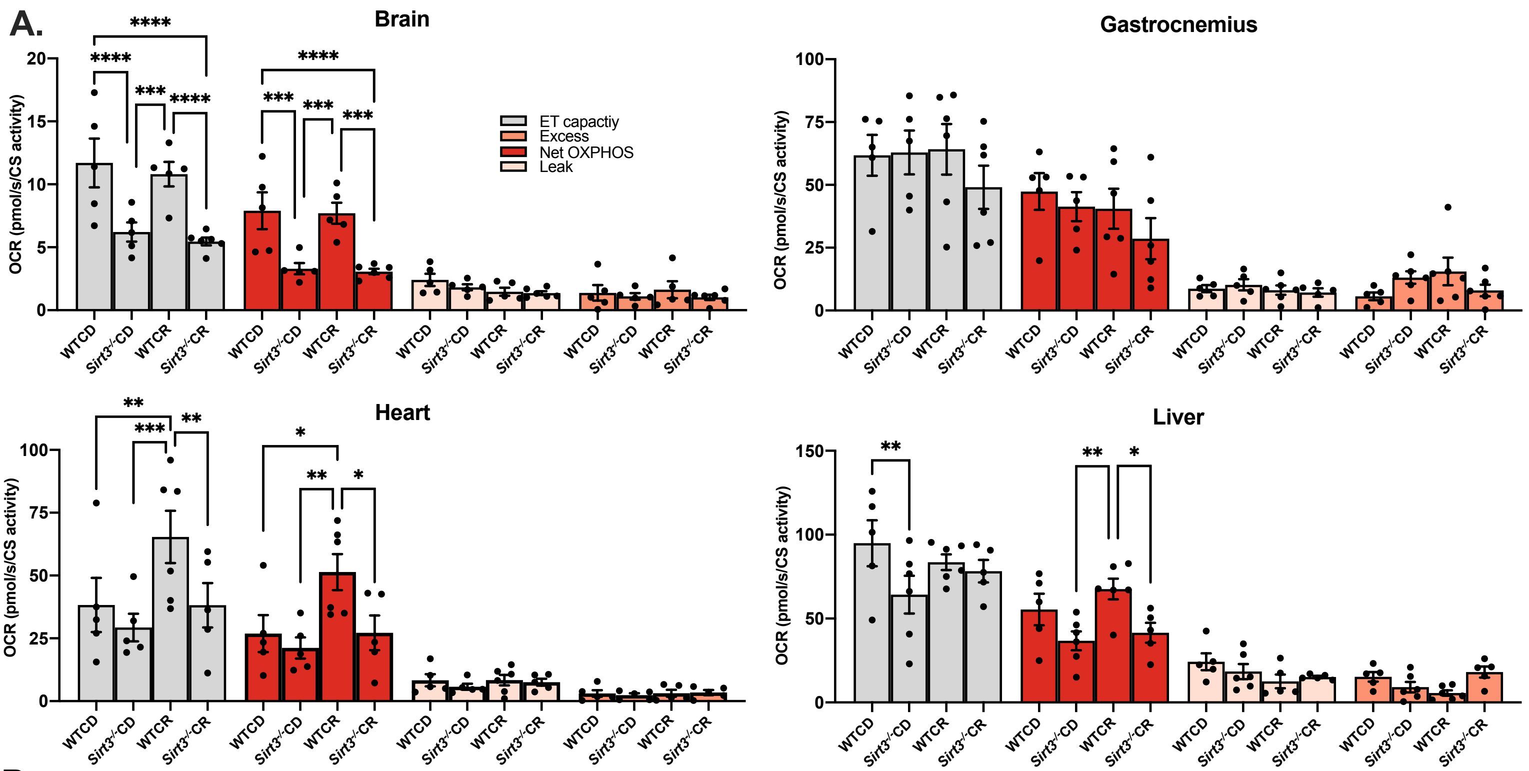

B.

Brain

Leak		
Sirt3$^{-/-}$CD vs. WTCR	*	*p*=0.0143
**Net OXPHOS**		
WTCD vs. *Sirt3*$^{-/-}$CD	*	*p*=0.0422
Sirt3$^{-/-}$CD vs. WTCR	**	*p*=0.0028
WTCR vs. *Sirt3*$^{-/-}$CR	*	*p*=0.0133

Gastrocnemius

Net OXPHOS		
WTCD vs. *Sirt3*$^{-/-}$CR	*	*p*=0.0169

Heart

Leak		
WTCD vs. WTCR	*	*p*=0.0464
WTCR vs. *Sirt3*$^{-/-}$CR	*	*p*=0.028
**Net OXPHOS**		
WTCD vs. WTCR	*	*p*=0.0449
Sirt3$^{-/-}$CD vs. WTCR	*	*p*=0.0442
WTCR vs. *Sirt3*$^{-/-}$CR	*	*p*=0.0242

Liver

Net OXPHOS		
WTCD vs. WTCR	**	*p*=0.0023
Sirt3$^{-/-}$CD vs. WTCR	**	*p*=0.0025
WTCR vs. *Sirt3*$^{-/-}$CR	****	*p*<0.0001

C.

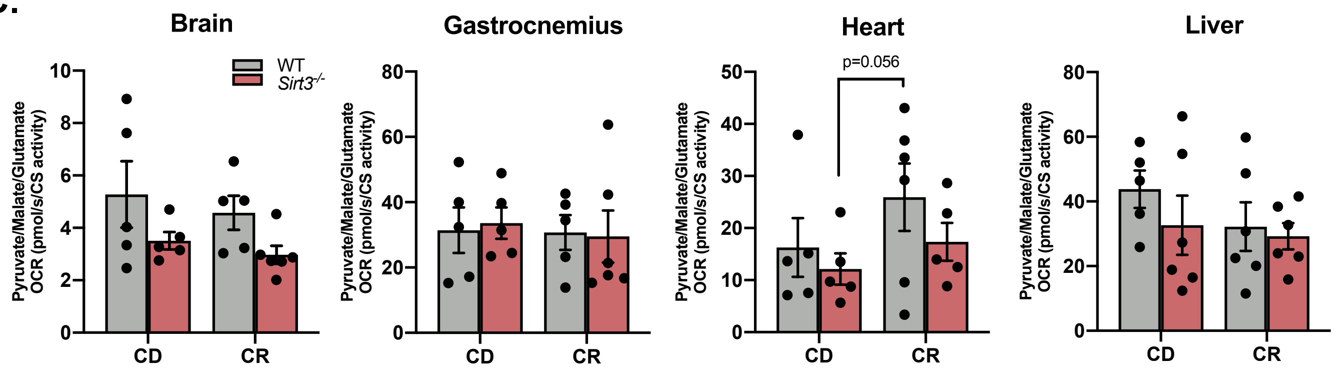

Fig S3.

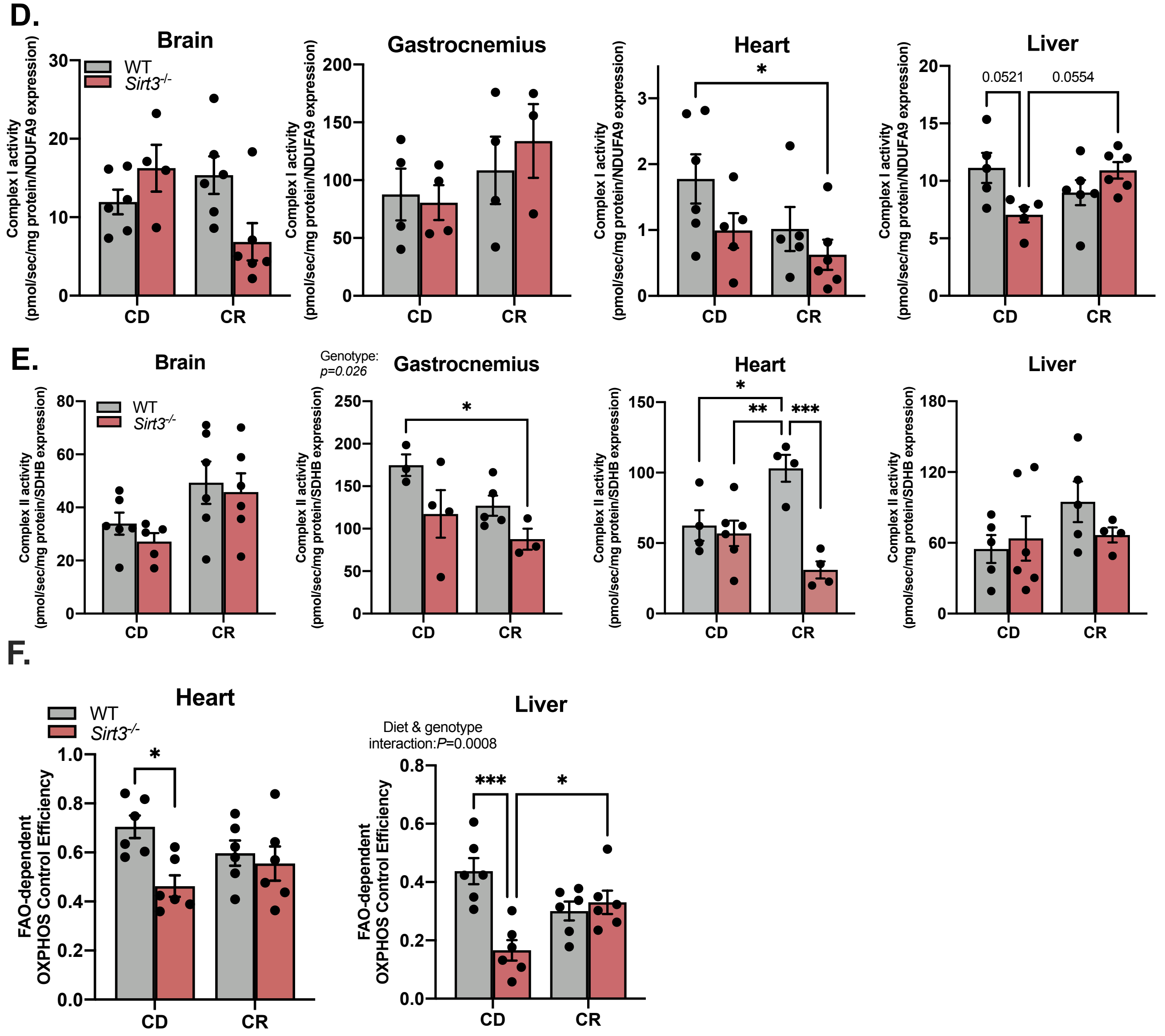

Fig S3.

A) Mitochondrial electron transfer (ET) capacity, net OXPHOS, leak and excess respiration of permeabilized brain, gastrocnemius, heart and liver from 25-month-old WTCD, *Sirt3*$^{-/-}$CD, WTCR, *Sirt3*$^{-/-}$ CR mice, n= 5-6 per group. Each of these respiration parameter was assessed by oxygen consumption rate (OCR). Plotted data were corrected to citrate synthase (CS) activity. These respiration parameters were assessed by oxygen consumption rate (OCR). Electron transfer capacity was assayed using pyruvate, glutamate, malate, succinate as substrates upon mitochondrial uncoupler FCCP addition. Leak respiration was assayed using pyruvate, glutamate, malate in the absence of ADP. Coupled respiration was assayed using pyruvate, glutamate, malate, succinate in the presence of ADP. Net OXPHOS respiration was calculated by subtracting leak respiration from coupled respiration. Excess respiration is calculated by subtracting coupled respiration from electron transfer capacity.

B) Statistics table of Fig. 3B.

C) NADH-linked coupled respiration was assessed using pyruvate, glutamate, malate as substrates in the presence of ADP in brain, gastrocnemius, heart and liver from 25-month-oldtreatment groups, n= 5-6 per group.

D-E) Complex I, II enzymatic activity of brain, gastrocnemius, heart and liver from 25-month-old treatment groups. Complex activity was normalized to total protein and NDUFA9 (Complex I) or SDHB (Complex II) protein expression. n= 3-6 per group for Complex I activity and n= 3-6 per group for Complex II activity.

F) FAO-dependent net OXPHOS control efficiency was obtained by (coupled FAO respiration – leak FAO respiration) /(coupled FAO respiration), where coupled FAO respiration was assessed using palmitoylcarnitine, malate as substrates in the presence of ADP and leak FAO respiration was assessed using palmitoylcarnitine, malate as substrates in the absence of ADP.

Data were analyzed by two-way ANOVA followed by multiple t-tests. *p* value reported for each comparison is corrected by Tukey's test. Results plotted as mean ± SEM. *: $p \leq 0.05$; **: $p \leq 0.01$; ***: $p \leq 0.001$; ****: $p \leq 0.0001$. Significant ($p \leq 0.05$) diet effect, genotype effect and/or diet and genotype interaction for each experiment are indicated in figures.

Fig S4.

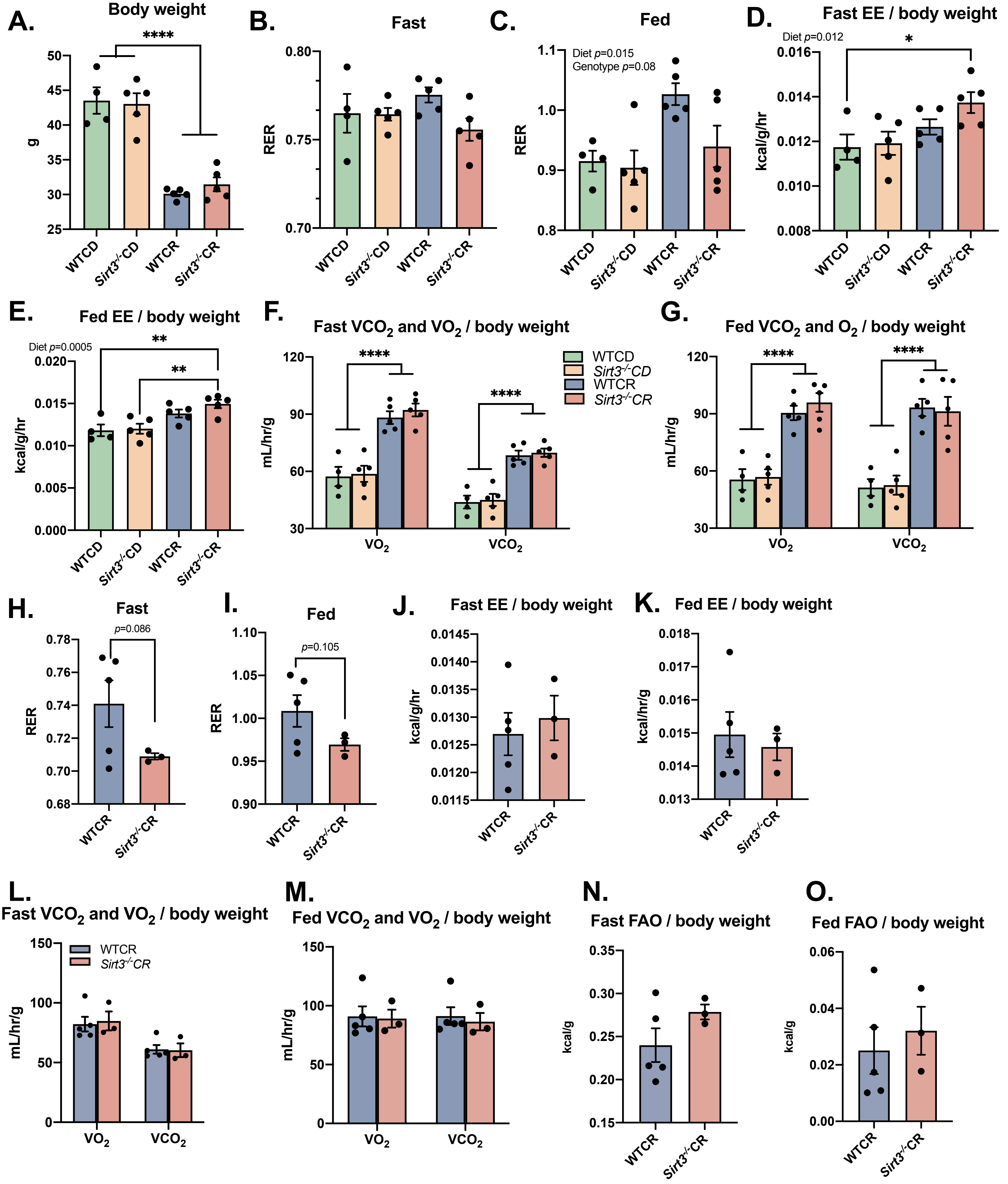
A.
Body weight
****
g
WTCD
$Sirt3^{-/-}$CD
WTCR
$Sirt3^{-/-}$CR
B.
Fast
RER
C.
Fed
Diet p=0.015
Genotype p=0.08
RER
D.
Fast EE / body weight
Diet p=0.012
*
kcal/g/hr
E.
Fed EE / body weight
Diet p=0.0005
**
**
kcal/g/hr
F.
Fast VCO_2 and VO_2 / body weight
****
****
mL/hr/g
WTCD
$Sirt3^{-/-}$CD
WTCR
$Sirt3^{-/-}$CR
VO_2
VCO_2
G.
Fed VCO_2 and O_2 / body weight
****
****
mL/hr/g
H.
Fast
p=0.086
RER
I.
Fed
p=0.105
RER
J.
Fast EE / body weight
kcal/g/hr
K.
Fed EE / body weight
kcal/hr/g
L.
Fast VCO_2 and VO_2 / body weight
WTCR
$Sirt3^{-/-}$CR
mL/hr/g
M.
Fed VCO_2 and VO_2 / body weight
mL/hr/g
N.
Fast FAO / body weight
kcal/g
O.
Fed FAO / body weight
kcal/g

Fig S4.

P.

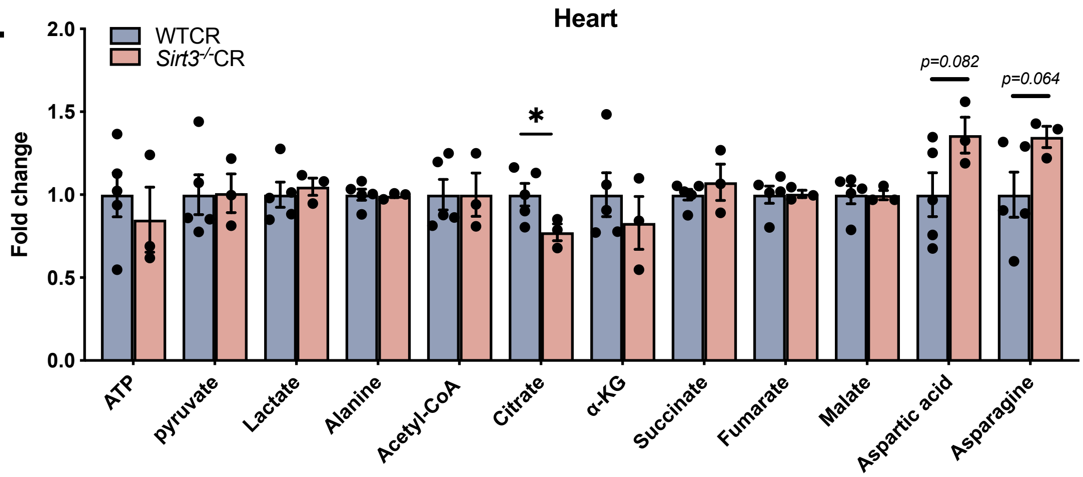
Heart
WTCR
Sirt3-/-CR
Fold change
p=0.082
p=0.064
*
ATP
pyruvate
Lactate
Alanine
Acetyl-CoA
Citrate
α-KG
Succinate
Fumarate
Malate
Aspartic acid
Asparagine

Q.

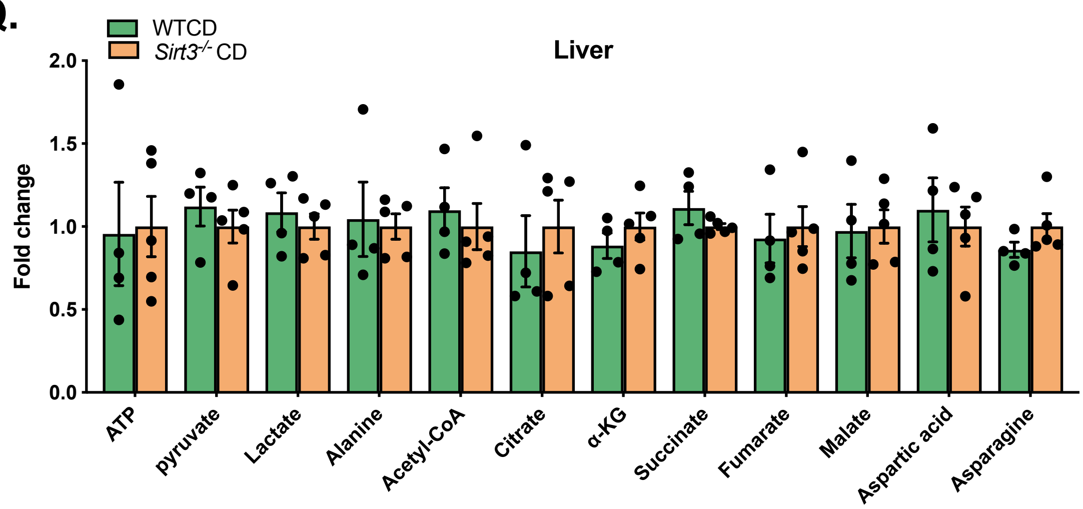
Liver
WTCD
Sirt3-/- CD
Fold change
ATP
pyruvate
Lactate
Alanine
Acetyl-CoA
Citrate
α-KG
Succinate
Fumarate
Malate
Aspartic acid
Asparagine

R.

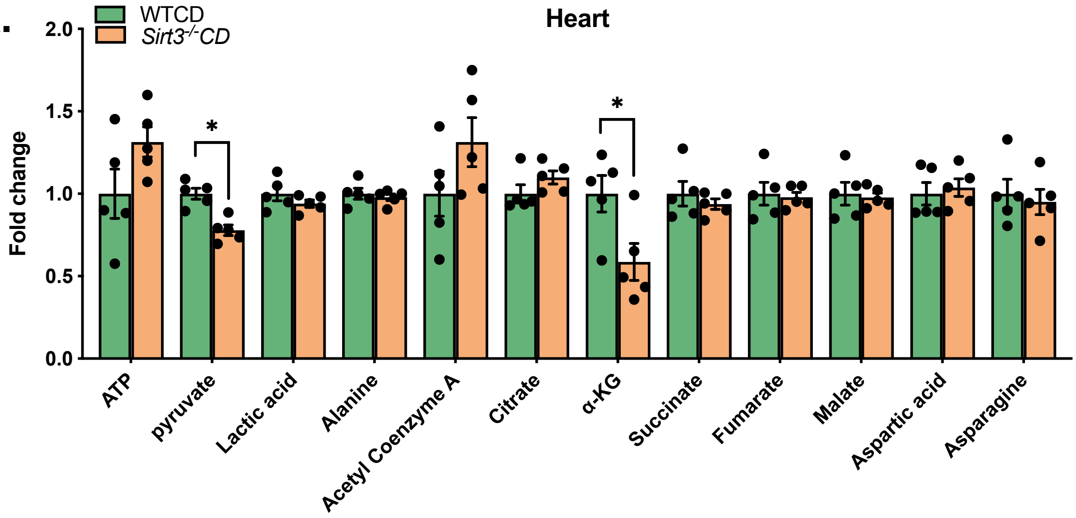
Heart
WTCD
Sirt3-/-CD
Fold change
*
*
ATP
pyruvate
Lactic acid
Alanine
Acetyl Coenzyme A
Citrate
α-KG
Succinate
Fumarate
Malate
Aspartic acid
Asparagine

Fig S4.

S.

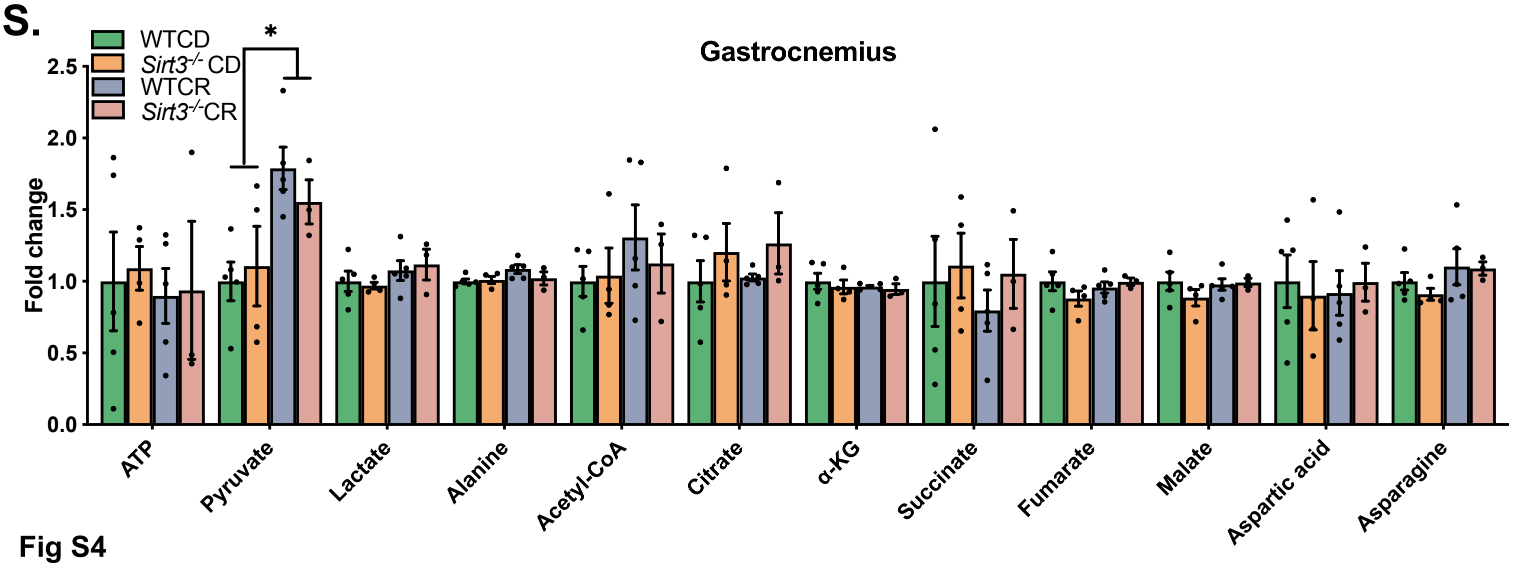

Fig S4

A-G. Metabolic chamber study of 25-month-old WTCD (n=4), *Sirt3*$^{-/-}$CD (n=5), WTCR (n=5) and *Sirt3*$^{-/-}$ CR mice (n=5). Experiment consisted of a 24-hour fasting period followed by a refeeding period with 8 hours of food provision (Fig. 4A,B).

A) Body weight of 4 groups of mice.

B-C) RER of fast and fed states, calculated through averaging RER data points of individual mouse for each state.

D-E) Energy expenditure per hour during fast and fed states, corrected to individual mouse body weight.

F-G) Volume of CO_2 generated and O_2 consumed per hour during fast and fed states, corrected to individual mouse body weight.

Data were analyzed by two-way ANOVA followed by multiple t-tests. *p* value reported for each comparison is corrected by Tukey's test. Results plotted as mean ± SEM. *: p≤0.05; **: p≤0.01; ***: p≤0.001; ****: p≤0.0001. Significant (p≤0.05) diet effect, genotype effect and/or diet and genotype interaction for each experiment are indicated in figures.

H-O. Metabolic chamber study of 25-month-old WTCR (n=5) and *Sirt3*$^{-/-}$ CR mice (n=3). Experiment consisted of a 24 hours of fasting followed by a refeeding period in which the normal daily food allotment used in the lifespan study was provided, and no food was removed during the experiment.

H-I) RER of fast and fed states, calculated through averaging RER data points of individual mouse for each state.

J-K) Energy expenditure per hour during fast and fed states, corrected to individual mouse body weight.

L-M) Volume of CO_2 generated and O_2 consumed per hour during fast and fed states, corrected to individual mouse body weight.

N-O) FAO during fast and fed states, corrected to individual mouse body weight.

Data were analyzed unpaired Welch's t-test. Results plotted as mean ± SEM.

P-S) Fold change of major TCA metabolites in 25-month-old mice after a 6-hour refeeding, n=3-5.

Fig. S4P-R were analyzed by Welch t-test. Results plotted as mean ± SEM. *: p≤0.05. Each metabolite in Fig. S4S was analyzed by two-way ANOVA followed by multiple t-tests. *p* value reported for each comparison is corrected by Tukey's test. Results plotted as mean ± SEM.
