# Supplementary material for: SIRT3 deficiency decreases oxidative metabolism capacity but increases lifespan in male mice under caloric restriction
Source: Aging Cell. 2022 Oct 5;21(12):e13721. doi: 10.1111/acel.13721 (PMC9741511; doi:10.1111/acel.13721)
Supplement: Supplementary file 2 — Appendix S1 [file ACEL-21-e13721-s001.docx]

**Supplemental Material**

**Methods**

**SIRT3 deficiency decreases oxidative-metabolism capacity but increases lifespan in male mice under caloric restriction**

Rashpal S Dhillon^1,2*^, Yiming (Amy) Qin^1,2,3*^, Paul R van Ginkel^4^, Vivian X Fu^4^, James M Vann^4^, Alexis J Lawton^1,2^, Cara L Green^5,6^, Fúlvia B Manchado-Gobatto^7^, Claudio A Gobatto^7^, Dudley W Lamming^3,5,6^, Tomas A Prolla^4,8^, John M Denu^1,2,3,8^

^1^ Department of Biomolecular Chemistry, University of Wisconsin-Madison, Madison, WI, USA;

^2^ Wisconsin Institute for Discovery, University of Wisconsin-Madison, Madison, WI, USA;

^3^ Interdisciplinary Graduate Program in Nutritional Sciences, University of Wisconsin-Madison, Madison, WI, USA;

^4^ Department of Genetics and Medical Genetics, University of Wisconsin-Madison, Madison, WI, USA.

^5^ Department of Medicine, SMPH, University of Wisconsin-Madison, Madison, WI, USA;

^6^ William S. Middleton Memorial Veterans Hospital, Madison, WI, USA;

^7^School of Applied Sciences, ﻿Laboratory of Applied Sport Physiology, University of Campinas, Limeira, Brazil

^8^Lead Contact

*These authors contributed equally to this work

Correspondence: [john.denu@wisc.edu](http://john.denu@wisc.edu) and [taprolla@wisc.edu](mailto:taprolla@wisc.edu)

John M. Denu, Ph.D.

Professor of Biomolecular Chemistry

University of Wisconsin-Madison

330 North Orchard Street

Madison, WI, 53715

[john.denu@wisc.edu](mailto:john.denu@wisc.edu)

608-316-4341

Tomas A. Prolla, Ph.D.

Professor of Genetics and Medical Genetics

University of Wisconsin-Madison

3430 Genetics, 425-g Henry Mall

Madison, WI 53706-1580

taprolla@wisc.edu

608-265-5204

**Key words:** calorie restriction, lifespan, mitochondrial acetylation, mitochondrial respiration, fatty acid oxidation, aerobic fitness, fuel switching, pseudo-fasting

Rashpal S Dhillon, [dhillon@zoology.ubc.ca](mailto:dhillon@zoology.ubc.ca); Yiming (Amy) Qin, [qin26@wisc.edu](mailto:qin26@wisc.edu); Paul R van Ginkel, [prvangin@wisc.edu](mailto:prvangin@wisc.edu); Vivian X Fu, [vxfu@wisc.edu](mailto:vxfu@wisc.edu); James M Vann, [jmvann@wisc.edu](mailto:jmvann@wisc.edu); Alexis J Lawton, [alawton2@wisc.edu](mailto:alawton2@wisc.edu); Cara L Green, [cara.green@wisc.edu](mailto:cara.green@wisc.edu); Fúlvia B Manchado-Gobatto, [fgobatto@unicamp.br](mailto:fgobatto@unicamp.br); Claudio A Gobatto, [claudio.gobatto@fca.unicamp.br](mailto:claudio.gobatto@fca.unicamp.br); Dudley W Lamming, [dlamming@medicine.wisc.edu](mailto:dlamming@medicine.wisc.edu); Tomas A Prolla, [taprolla@wisc.edu](mailto:taprolla@wisc.edu); John M Denu, [john.denu@wisc.edu](mailto:john.denu@wisc.edu) .

**EXPERIMENTAL MODEL AND SUBJECT DETAILS**

All animal studies were conducted at the AAALAC-approved Animal Facilities at the William S. Middleton Memorial Veterans Administration Medical Center and the University of Wisconsin-Madison under animal research protocols approved by these institutions Institutional Animal Care and Use Committee (IACUC).

**Animals**

Male and female *Sirt3^+/−^* mice (#011664-UNC) were purchased from the Mutant Mouse Resource Centers (MMRRC) at the University of North Carolina-Chapel Hill (Chapel Hill, NC). These mice were created by a retroviral promoter trap that functionally inactivates one allele of the Sirt3 gene by a 5.1 kb retroviral insertion in the intron preceding coding exon 1 (MGI:3529767). *Sirt3^+/+^* C57BL/6J mice were purchased from The Jackson Laboratory (Stock number 000664). *Sirt3^+/−^* mice were then backcrossed onto the C57BL/6J background (Someya *et al.*, 2010). To obtain functional NNT in *Sirt3* mice, *Sirt3^-/-^* C57BL/6J male mice were interbred with female *Sirt3^+/+^* C57BL/6NJ (Nnt^+/+^) mice (The Jackson Laboratory, stock number 005304) producing (C57BL/6NJ x C57BL/6J) F1/Nnt^+/-^/Sirt3^+/-^ (abbreviated B6NJB6JF1/Nnt^+/-^/Sirt3^+/-^). These were interbred to produce the two genotypes of male mice, *Nnt^+/+^*/*Sirt3^-/-^* and wild-type counterparts *Nnt^+/+^*/*Sirt3^+/+^* used in experiments.

**Genotyping**

Genotyping for *Nnt* and *Sirt3* were accomplished using PCR reactions. DNA was prepared from tail snips obtained as mice were weaned at 3 weeks of age. The *Sirt3* genotyping reaction consisted of final concentrations of 1X reaction buffer and 0.05 U/ul Taq polymerase (Denville, Metuchen, NJ), 0.20 µM each primer (IDT), 0.20 mM dNTP mix and 1.75 mM MgCl_2_ (Thermo-Fisher/Invitrogen). *Sirt3^+/+^* mice were indicated by a 404 bp product across the insertion points of the (absent) viral insert. *Sirt3^-/-^* mice were indicated by two amplification products: 207 bp across the 5’ insertion site of the (present) viral insert and 291 bp across the 3’ insertion site. Heterozygous mice were indicated by the presence of all three bands. The *Nnt* genotyping reaction consisted of final concentrations of 1X reaction buffer and 0.10 U/ul Taq polymerase (Denville, Metuchen, NJ), 0.40 µM each primer (IDT), and 0.20 mM dNTP mix (Thermo-Fisher/Invitrogen). *Nnt^+/+^* mice were indicated by a 304 bp product across the deletion point of the mutation, which confirmed there was no deletion. *Nnt^-/-^* mice were indicated by two amplification products: 227 bp across the 5’ deletion site and 203 bp across the 3’ deletion site. Heterozygous mice were indicated by the presence of all three bands.

**Husbandry and Diets**

Starting at two months of age, male *Sirt3^-/-^* and *Sirt3^+/+^* mice were individually housed (12 hours light/dark cycle), randomly assigned to a control diet (CD) or calorie restricted diet (CR). Both CD and CR mice maintained their assigned, fixed calorie diet till death. Both control and CR groups were fed with food pellets that are a modified form of AIN-93M (Bio-Serve). The control group was fed 89 kcal/week of diet AIN-93M (Bio-Serve, Farmington, NJ), which is ~16% less than the average ad lib intake 105.8 kcal/week (Pugh, Klopp and Weindruch, 1999). The CR group was fed 67 kcal/week. The restricted diet food pellets are a modified form of AIN-93M (Bio-Serve) and are nearly isocaloric gram-for-gram with the control diet food pellets, but enriched in protein, vitamins and minerals to provide the same amounts of these components as control diet mice receive. CD and CR mice were fed on Monday/ Wednesday/Friday at approximately 7:00 a.m. (CD mice: 7 grams on Monday/Wednesday, and 10 grams on Friday; CR mice: 5 grams on Monday/Wednesday, and 8 grams on Friday).

Any mice that appear ill, cachectic, stressed, less than 22 grams indicated by cage card were euthanized. As these mice age, further criteria for euthanasia included: body weight loss of 20% in one month, and persistent severe ulcerative dermatitis. Mice that have a body condition score less than 1.5 on the Ullman-Cullere and Foltz Body Condition Scale (Ullman-Culleré and Foltz, 1999) were euthanized.

**Survival Study**

All animals were inspected each day. Mortality during the survival study was analyzed using the log-rank test to compare the differences in Kaplan-Meier survival curves. Median lifespan and maximal lifespan were defined as the time of 50% mice survival and the average lifespan of top 10% longest lived mice. Maximum lifespan comparison was performed using two-way ANOVA followed by multiple t-test, corrected by Tukey’s test. Maximum lifespan statistics was also confirmed with Boschloo's Test. Graph Pad Prism 9.1.1 was used for visualization and OASIS-2 (Han *et al.*, 2016) was used for Log-rank test and Boschloo's Test.

**Body Weight and Composition**

Two-month-old mice, immediately after entering CR, were weighed weekly. Exceptions were made when weight decreased by one gram (or ~5% body weight) in one week, in which case the mice were weighed on the same days as they were fed until they gained weight. If a mouse lost 4 grams (or ~20% body weight) in one week, it was euthanized. Adult CR mice (>6 months) were weighed weekly as long as their weight was at or above 25 grams. If their weight was between 22-25 grams, they were then weighed on the same days as feeding. If mice weighed less than 22 grams, or their body condition score decreased to 2.0 or less, they were weighed daily. The control group of mice were weighed once per month. Control mice were then weighed once every two weeks starting at age 24 months and weighed weekly staring at age 29 months (or 50% mortality rate was reached). Mouse body composition was determined using the EchoMRI Body Composition Analyzer (EchoMRI, Houston, TX, USA).

**Transmission Electron Microscopy**

Tissue for transmission electron microscopy (TEM) was sampled from muscle fibers collected from the gastrocnemius and heart left ventricular wall. Tissue samples were fixed and mounted at the Medical School Electron Microscope Facility (University of Wisconsin-Madison). Briefly, samples were fixed in 2.5% glutaraldehyde in 0.1 M phosphate buffer, post-fixed in 1.0% osmium tetroxide, and dehydrated in a graded series of ethanol concentrations (30-100%), prior to embedding in epoxy resin. Ultrathin (0.5 µm) sections were then mounted for post fixing in uranyl acetate and lead citrate. Sections were viewed using a Philips CM120 transmission electron microscope and images were captured with a BioSprint 12 series digital camera. Four mice per treatment group at 25 months of age were analyzed. Measurements were taken at a final magnification of 5600x and 19500x for mitochondrial volume density and morphology, respectively. Volume density was calculated using the summed point count method (Schmiedl *et al.*, 1990). This method uses the number of intersections of a grid landing on mitochondria relative to those intersections landing on the reference space as an index of volume density. TEM samples were analyzed blindly by preparing samples with a new set of numbers and cross referenced after analysis to remove any experimenter bias during the measurement process.

**Tissue Homogenate Preparation**

Pulverized tissue was suspended in hypotonic buffer (﻿25 mM K_2_HPO4, 5 mM MgCl_2_, pH 7.2) with protease inhibitor cocktail (100X, Thermo Scientific). The mixture was then sonicated for three 10-second cycles (10 seconds pauses between each cycle, 30% amplitude) and centrifuged at 1,000xg for 10 mins at 4˚C. Supernatant was transferred to a new tube and centrifuged again at 1,000xg for 10 mins at 4˚C. Total protein concentration of the supernatant was determined using Pierce BCA protein assay kit (Thermo Scientific). Supernatant was aliquoted and stored at -80˚C.

**Citrate Synthase Activity**

Citrate synthase activity assay was described by Hepple *et al*., (2005). Briefly, citrate synthase activity was determined by measuring the production rate of TNB (thionitrobenzoic acid) at an absorbance of 412nm. To initiate the reaction, reaction buffer (final concentration: 0.3mM acetyl-CoA, 100mM Tris buffer pH 8.0, 0.1mM DTNB, and 0.5mM oxaloacetate) was added into properly diluted tissue homogenate. Absorbance at 412nm was measured using a plate reader (Synergy™ H4 Hybrid Multi-Mode Microplate Reader) at 37˚C. Citrate synthase activity was normalized to the amount of total protein in each sample.

**Mitochondrial DNA Content**

Methods to determine the copy number of mitochondrial DNA per nucleus is described by Patil *et al.*, (2015). Intronic sequences close to those used by Patil *et al*., (2015) for LPL (NC_000074.6) and Nd1 (NC_005089.1). A multiplex PCR was designed using a FAM-labeled probe for LPL and a HEX-labeled probe for Nd1. Both probes incorporated the internal ZEN™ dark quencher in addition to the 3’ quencher Iowa Black^®^ FQ.

A gBlocks® Gene Fragment (IDT) served as a standard curve template for both LPL and Nd1 to determine copy number. The oligonucleotide template includes 246 bp of the LPL sequence and 213 bp of the Nd1 sequence. Standards included with each PCR run were 0, 10, 100, 1,000, 10,000, 33,000, 100,000, and 1 million copies of the template. RNA and DNA were prepared by an adaptation of the method of Hofer *et al.*, (2006). Approximately 50 mg of tissue was homogenized using a Tissuelyzer II (Qiagen) at 25 cycles/second for two runs of 30 seconds each in GTC buffer (3M guanidine thiocyanate, 0.2%lauroylsarcosinate, 20 mM Tris buffer, pH 7.5) made to 20 mM deferoxamine mesylate immediately before use. An equal volume of phenol-chloroform-isoamyl alcohol (25:24:1) pH 6.7 was added, vortexed immediately and repeatedly vortexed during a 10-minute incubation at room temperature. Samples were centrifuged at 14,000xg at 4°C for 10 min. Then the upper aqueous layer was removed, mixed with an equal volume of isopropanol, and precipitated at -20°C for one hour. Samples were then centrifuged at 14,000xg at 4°C for 10 min and washed twice with 70% ethanol. The pellet containing RNA and DNA was dried and re-dissolved in 50 µl of chilled nuclease-free water. DNA was separated from RNA using the Macherey-Nagel Nucleospin Tri-prep spin column kit according to the manufacturer’s instructions (Macherey-Nagel, Düren, Germany). RNA and DNA were then re-precipitated in 70% ethanol, 0.83 M sodium acetate and washed twice with 70% ethanol. Samples were re-dissolved in 50 µl of TE buffer. After initial reading of DNA concentrations in a Nanodrop 2000 (Thermo-Fisher) samples were diluted to approximately 7.5 ng/µl. Concentrations were measured again using a Quant-IT Fluorescent assay (Thermo-Fisher). These final concentration values were used to normalize the PCR results.

PCR reactions consisted of PrimeTime Gene Expression Master Mix (IDT), LPL primers and FAM-labelled probe, Nd1 primers and HEX-labelled probe and 2 µl of sample containing approximately 15 ng of sample DNA in a final reaction volume of 10 µl. Real-time PCR was performed in an Eppendorf Realplex Thermocycler with an initial denaturation step of 95°C for 3 min, then 40 cycles of 95°C for 5 seconds and 60°C for 30 seconds. Cycle thresholds of the standards were used to generate a standard curve to calculate the copies of mtDNA and genomic DNA in each sample.

**Automated Capillary Electrophoresis Based Immunoblot**

All proteins analyzed by automated capillary electrophoresis-based immunodetection system (WES, ProteinSimple) were detected using the 12-230kDa WES separation module (SM-W004, ProteinSimple) and a detection module (depending on primary antibody source, an anti-rabbit (DM-001) or anti-mouse (DM-002) detection module was used). Briefly, tissue lysates in RIPA buffer were diluted in 0.1x sample buffer (optimized per tissue in preliminary experiments) and 8µl was combined with 2µl 5x fluorescent master mix. Samples were denatured by heating at 95˚C for 5 minutes (50˚C, 5 minutes for detection of ETC proteins). For immunodetection, blocking buffer, primary antibody (in per tissue optimized dilution), secondary antibody, chemiluminescent substrate, samples (including a biotinylated size marker) and wash buffer were loaded in in the above order in the designated wells on the supplied microplate. The plate was centrifuged for 5 mins at 1,000xg to remove air bubbles and loaded into WES. Default separation parameters were used for single protein detection. For ETC Complex IV and multiplexing of ETC Complex III and V specific antibodies, sample loading time and separation time were increased to 21mins and 31mins, respectively. Following automated protein separation and detection, data were analyzed using Compass for SW software (version 3.1.7, ProteinSimple) and the area under the specific antibody peak was measured. To compare protein expression among samples of a tissue type, expression was normalized to total protein content. Total protein was analyzed in all the samples in a separate set of assays using a biotin-based protein labeling and detection system (DM-RP01) on the WES. Total protein was also analyzed using Compass for SW software as total area under the peaks. Two major peaks that were determined to result from direct binding of the biotin detection reagent to protein were excluded from analysis. Control carryover samples between plates were used to correct for inter-plate signal intensity variability (generally not exceeding 10%). The area under the specific antibody peak in each sample was divided by the area under the total protein peaks of that sample and averaged among the same tissue samples from the mice of the same genotype on the same diet (n=6). Primary antibodies were used to detect Citrate Synthase (#14309, CST), SIRT3 (#5490, CST), NDUFA9 (ab14713, abcam), SDHB (ab14714, abcam), UQCRC2 (ab14745, abcam), MTCOI (ab14705, abcam) and ATP5A (ab14748, abcam).

**Mitochondria Isolation**

Freshly harvested tissue was quickly rinsed in 1xPBS and placed in an ice-cold petri-dish with isolation media (250mM sucrose, 25 mM KH_2_PO_4_, 50 mM KCl, 10 mM HEPES, 0.5 mM EGTA, 1% BSA, pH 7.4). Tissue was finely minced and transferred to an ice-cold glass homogenizer. Tissue was homogenized with a tight pestle on ice. The number of strokes was optimized for each tissue. Homogenate was then transferred into a conical tube and centrifuged at 900xg for 10 mins at 4˚C. Supernatant was filtered through a layer of glass wool and centrifuged at 9,000xg for 10 mins at 4˚C. Supernatant was discard, and the mitochondrial pallet was resuspended in isolation media before being centrifuged again at 9,500xg for 10 mins at 4˚C. The supernatant was again discarded, and the mitochondrial pellet was suspended in storage buffer (25 mM KH_2_PO_4_, 50 mM KCl, 10 mM HEPES, 0.5 mM EGTA, pH 7.4) and stored at -80˚C.

**Western Blot**

Pulverized tissues or isolated mitochondria were lysed in RIPA buffer (50mM Tris-HCl, 150mM NaCl, 0.1% Triton-100, 0.5% sodium deoxycholate, 0.1% SDS, 1mM EDTA, protease inhibitor cocktail (100X, Thermo Scientific), 10mM nicotinamide and 10mM sodium butyrate). Tissue lysates were sonicated for three 5-second cycles (10 seconds pauses between each cycle, 20% amplitude) and mitochondrial lysates were agitated for 1 hours at 4˚C. Lysates were centrifuged at 12,000rpm for 20 mins at 4˚C, and the supernatants were placed into new tubes. Total protein concentration of each sample was determined by Pierce BCA protein assay kit (Thermo Scientific). Each sample, containing ~30µg total protein, was mixed with loading buffer (4X, LI-COR) and heated for 5 mins at 95˚C. These samples were separated on 10% in-house casted SDS-Page gels and transferred onto nitrocellulose membrane (GE). Total protein stain (LI-COR) was used for total protein normalization. After washing for 10 mins in TBST (20mM Tris, pH7.5, 150mM NaCl, 0.1% Tween-20), membranes were blotted in 5% BSA in TBST at room temperature for 1 hour and incubated overnight with primary antibody (in 2% BSA TBST) solution at 4˚C. The next day, membranes were washed with TBST and incubated with secondary antibody (LI-COR, 1:10,000) for 90 mins at room temperature. Membranes were washed for 5 minutes in TBST 3 times and followed by two 5-min TBS wash. Membranes were imaged using the Odyssey scanner (LI-COR Odyssey). Images were further analyzed by Image Studio Lite version 5.2.5 (LI-COR). Western blot primary antibodies used include: SIRT3 (#5490, CST, 1:1000), Acetylated-Lysine (#9681, CST, 1:1000), VDAC (75-204, NeuroMab, 1:1250).

**LC-MS Based Quantification of Mitochondrial Acetylation Stoichiometry**

Quantification of acetylation stoichiometry follows methods described previously in Baeza *et al.*, 2020, with the following modifications. Notably, the current study quantified acetylation stoichiometry of individual lysine residues using an antibody free approach, which does not involve acetyl-peptide immunoprecipitation step during sample preparation. Analysis was conducted using 200 μg of protein from isolated mitochondrial subcellular fractions, which were denatured in urea buffer (6 M urea (deionized), 100 mM ammonium bicarbonate pH = 8.0, 5 mM DTT). Samples were incubated for 20 minutes at 60 °C, then cysteines were alkylated with 50 mM iodoacetamide and incubated for 20 minutes. Chemical acetylation using two rounds of ~20 µmol heavy isotopic D6-acetic anhydride (Cambridge Isotope Laboratories). Samples were diluted using 100 mM ammonium bicarbonate pH = 8.0 to 2 M urea and digested with 1:100 trypsin at 37 °C for 4 hours. Samples were then diluted to 1 M urea prior to a second digestion by gluC (1:100). Chemically acetylated peptides were fractionated into 6 fractions using a Shimadzu LC-20AT HPLC system with a Phenomenex Gemini^®^ NX-C18 column (5µm, 110Å, 150 x 2.0mm). The samples were analyzed using data-independent acquisition (DIA) analysis by a Thermo Q-Exactive Orbitrap coupled to a Dionex Ultimate 3000 RSLC nano UPLC with a Waters Atlantic reverse phase column (100 μm x 150 mm).

To deconvolute and analyze the DIA spectra, a spectral library containing all light and heavy acetyl-lysine feature pairs was generated. Spectral library samples were processed identically to the experimental samples, except that they were treated with C12-acetic anhydride (Sigma) and analyzed using data dependent acquisition (DDA) mass spectrometry analysis. A spectral library was generated using the openly available MaxQuant (v1.6.0) software package. Carbamidomethylation (C) was set as a fixed modification, and Oxidation (M) and Acetyl (K) were set as variable modifications. Trypsin and gluC were set as the digestion enzymes, with the max number of missed cleavages set to five. DDA runs from both the mitochondrial and cytosolic fractions were run to make one combined library. Heavy acetyl fragment ion pairs were generated in silico, such that the spectral library would contain both the light (endogenous) acetylation peaks and the heavy (chemical) acetylation peaks. The experimental samples were processed using Spectronaut (v10) using the generated spectral library. The subcellular fraction experimental samples were processed separately. The data was processed and cleaned using an in-house R script, which can be accessed through the GitHub link: [DOI:10.5281/zenodo.3360892], such that stoichiometry was calculated from the ratio of endogenous (light) fragment ion peak area over the total (endogenous and chemical) fragment ion peak area. One-way ANOVA analyses were used to determine statistically significant differences in acetylation stoichiometry driven by age, diet, or genotype between two groups. The raw data, processed data, spectral library, and the analysis logs describing the settings for the Spectronaut analyses have been deposited to the ProteomeXchange Consortium via the MassIVE partner repository with the dataset identifier MSV000087085 and PXD024961 [doi:10.25345/C59Z2Q].

**Critical Velocity**

The critical velocity (CV) was determined according to the protocol previously described (Scariot *et al.*, 2019), based on the mathematical relationship between exercise intensity and time to exhaustion (tlim). Briefly, animals were subjected to four exhaustive running efforts applied on different days, with the time to exhaustion recorded for each exercise intensity. Before the actual experiment, mice were adapted to a treadmill ergometer (Exerc 3/6 Treadmill, Columbus Instruments, Ohio, USA) and to exercise for three days (5 min per day). To determine CV, constant exercise intensities (treadmill velocity ranging from 7.5 to 20.0 m/min) were individually selected so that the time to exhaustion was no more than 15 min and no less than 1 min. The time to exhaustion, recorded in seconds, was determined when the mouse was unable to run despite encouragement (gentle soft brush tapping, without electrical stimulus). Distance traveled for each mouse during individual tests was calculated by multiplying the velocity of the treadmill and time to exhaustion. Distance traveled and time to exhaustion, total four data points, were then plotted and fitted into a linear regression model. The goodness of the linear regression was evaluated by R^2^. The slope of the fitted regression corresponded to the critical velocity of the mouse.

**Mitochondrial Respiration in Permeabilized Tissues**

Brain (cerebral cortex), gastrocnemius fiber, heart and liver were freshly harvested from mice sacrificed by acute conscious cervical dislocation on the day of respiration experiments and temporally stored in iced cold BIOPS buffer (2.77 mM CaK_2_EGTA, 7.23 mM K_2_EGTA, 50 mM MES hydrate, 0.5 mM dithiothreitol, 20 mM imidazole, 20 mM taurine, 15 mM sodium phosphocreatine, 6.56 mM MgCl2, 5.77 mM ATP). Tissues were then permeabilized in BIOPS containing 30µg/ml saponin for 30 mins at 4˚C with gentle shaking (detailed tissue permeabilization steps were described in Doerrier *et al.*, (2018) and Kuznetsov *et al*., (2008). After being permeabilized, tissues were placed in cold Mir05 buffer (110 mM sucrose, 60 mM potassium lactobionate, 0.5 mM EGTA, 3 mM MgCl_2_, 20 mM taurine, 10 mM KH_2_PO_4_, 20 mM HEPES, 1g/L BSA, pH 7.1) and stirred generally for 10 mins at 4˚C. Wet tissue weight was then collected, and tissue was place into the chamber. Mitochondrial respiration was determined by measuring oxygen consumption rate (OCR) using OROBOROS O2k (Oroboros Instruments) in Mir05 buffer at 37˚C. When oxygen consumption rate reached steady state, substrates were added in the following order with final concentration in 2ml chamber. 5mM pyruvate, 2mM malate, 10mM glutamate were added to establish leak respiration, followed by 1mM ADP to stimulate a coupled respiration rate. 10µM Cytochrome C was added to assess any outer membrane damage to the mitochondria. Then 10mM succinate was used to stimulate complex II-fueled respiration, followed by FCCP to measure uncoupled respiration. 0.5µM rotenone was used to isolate complex II respiration from complex I, and finally 2.5µM antimycin was used to estimate residual respiration. To examine fatty acid oxidation-dependent respiration, substrates were added in the following order with final concentration in 2ml chamber, 40 µM palmitoyl-carnitine with 0.1mM malate, 1mM ADP, 10µM Cytochrome C, FCCP, 2.5µM antimycin. Reported OCR for non-fatty acid substrates were normalized to CS activity, and OCR for non-fatty acid substrates were normalized to total protein.

**Mitochondrial Complex Activity**

Complex I and Complex II activity were determined by measuring the reduction of DCPIP with an absorbance of 600nm. Complex I activity was measured in triplicate by adding 250µl reaction mix A (hypotonic medium (25mM K_2_HPO_4_, 5mM MgCl_2_, pH7.2), 2.5mg/ml BSA, 100µM DCPIP, 65µM ubiquinone-2, 2µg/ml antimycin, 0.2mM NADH) to 5µl of properly diluted tissue homogenate (dilution was optimized for each tissue). Background was measured by adding 250µl reaction mix A with 5µM rotenone, to tissue homogenate. The reaction was monitored at an absorbance of 600nm for 20 mins. Complex II activity was measured in triplicate by adding 250µl reaction mix B (hypotonic medium (25mM K_2_HPO_4_, 5mM MgCl_2_, pH7.2), 2.5mg/ml BSA, 100µM DCPIP, 65µM ubiquinone-2, 2µg/ml antimycin, 2µg/ml rotenone, 20mM succinate) to 5µl of properly diluted tissue homogenate. Background was measured by adding 250 µl reaction mixture B, without succinate, to tissue homogenate. The reaction was monitored at an absorbance of 600nm for 20 mins. Reported Complex I and II activity were corrected to Complex I and Complex II expression, respectively, which were measured by WES as described in earlier sections.

**Metabolite Extraction and LC-MS-based Metabolite Profiling**

The metabolite extraction method was described in Haws *et al.* (2020) with the following modifications. Tissues were pulverized prior to metabolite extraction. Pulverized tissue (~30mg) was incubated with 1ml of ice cold 80:20 methanol: water solution for 5 mins on dry ice after 15-second vortexing. Tissue homogenate was centrifuged at maximum speed for 5 minutes at 4˚C. Supernatant was collected and transferred into a new tube. The remaining pellet was incubated with 400µl of ice cold 40:40:20 methanol:acetonitrile:water solution for 5 mins. Tissue homogenate was centrifuged again at maximum speed for 5 minutes at 4˚C. Supernatant was pooled with the first metabolite extraction. The 40:40:20 methanol:acetonitrile:water solution extraction was repeated once more and combined with the previous extractions. Three extractions were pooled and completely dried using SpeedVac (Thermo Fisher Savant ISS110) with nitrogen flow under room temperature. The dried metabolite samples were resuspended in water (150µl water per 5mg tissue) and centrifuged at maximum speed for 5 minutes at 4˚C. This supernatant was used for LC-MS metabolite profiling.

The metabolite detection method was adopted from Latorre-Muro *et al*. (2018). Briefly, metabolites were separated by Thermo Fisher Vanquish UHPLC with Waters Acquity UPLC BEH C18 column (1.7 μm, 2.1 × 100 mm; Waters Corp.) and analyzed by Thermo Fisher Q Exactive orbitrap mass spectrometer in negative ionization mode. LC separation was performed over a 25-minute method with a 14.5-minute linear gradient of mobile phase (buffer A, 97% water with 3% methanol, 10 mM tributylamine, and acetic acid-adjusted pH of 8.3) and organic phase (buffer B, 100% methanol) (0min, 5%B; 2.5min, 5%B; 17min, 95%B; 19.5min, 5%B; 20 min, 5%B; 25 min, 5%B, flow rate 0.2ml/min). 12µl of each sample was injected into the system for analysis. ESI settings were 30/10/1 for sheath/aux/sweep gas flow rates, 2.50 |kV| for spray voltage, 50 for S-lens RF level, 350°C for capillary temperature, and 300°C for aux gas heater temperature. MS1 scans were operated at resolution = 70,000, scan range = 85-1250 m/z, automatic gain control target = 1e6, and 100ms maximum IT. Metabolites were identified and quantified using El-MAVEN (v0.12.1-beta, Agrawal *et al.*, 2019) with metabolite retention times empirically determined in-house. Metabolite levels were compared using the peak AreaTop.

**Spontaneous Physical Activity**

The gravimetric method used to measure the spontaneous physical activity (SPA) was adopted from Biesiadecki *et al.* (1999) and Scariot *et al.* (2016) with modifications. In this study, the individual cage was put on the metallic platform equipped with one load cell (model PLA 10kgf, Lider Balanças, BR), capable of identifying the force generated by rodent activities. The instruments used to amplify and condition the signals were MKTC-05 (MK Controle e Instrumentação, BR) and NI-USB 6008 (National Instruments, USA), respectively. The signals were captured using digital acquisition software (LabVIEW SignalExpress National Instruments, USA) at 30Hz frequency. The gravimetric system was calibrated before the experiments by positioning known weights on the central point of the force platform. The signals obtained in volts were converted to units of gram using the linear regression equations (all calibrations presented R^2^ = 1.00) acquired from calibrations plots. Data were processed by using Matlab (R2008a MatLab, MathWork) as described in Scariot *et al.* (2019). In the current study, SPA, in arbitrary units, was registered during a 19-day period (including both light and dark phases, in all groups of 25-month-old mice).

**Quantitative PCR Analysis**

RNA was first isolated from ~30mg pulverized tissue using TRIzol Reagent (Invitrogen). Genomic DNA was removed using DNase I (Thermo Scientific) and cDNA was synthesized using RevertAid First Strand cDNA Synthesis Kit (Thermo Scientific) following manufacturer’s instructions. Then, the real-time PCR reactions were performed in triplicates using PerfeCTa SYBR Green SuperMix (Quantabio) and a Bio-Rad CFX96 C1000 Real-Time system. The expression levels of target genes were normalized against the housekeeping gene ﻿β2-microgulin (B2m).

Lcad, F: TCTTTTCCTCGGAGCATGACA, R: GACCTCTCTACTCACTTCTCCAG;

Etfdh, F: TCTTCCGATGAACAATCATGGC, R: AGTGGCGATTCCTTTTACACTAC;

Pparα, F: AGAGCCCCATCTGTCCTCTC, R: ACTGGTAGTCTGCAAAACCAAA;

Cpt1α, F: AGATCAATCGGACCCTAGACAC, R: CAGCGAGTAGCGCATAGTCA;

B2m, F: TTCTGGTGCTTGTCTCACTGA, R: CAGTATGTTCGGCTTCCCATTC.

**Whole Body Metabolic Assessment**

To measure metabolic parameters [O2, CO2, food consumption, respiratory exchange ratio (RER), energy expenditure], mice were acclimated to housing in a Oxymax/CLAMS metabolic chamber system (Columbus Instruments) and data from a continuous 48 h period was then recorded and analyzed. Mice were subjected to 2 hrs of acclimation prior to data collection. Although the baseline RER was steady after acclimation, we acknowledge that other metabolic parameters may take longer to reach the steady state in metabolic chamber vs. in normal environment condition. When cages were opened for addition/removal of food, data points were removed. RER, EE values were reported by the metabolic chamber system. FAO per hour were calculated by the equation: FAO (kcal/hr) = EE x (1-RER/0.3) (Bruss *et al.*, 2010). When calculated FAO <0, 0 was assigned to FAO for that hour. 24 hours FAO (kcal/24hr) was calculated from the 24-hr area under the curve of hourly FAO. Reported 24 hours FAO per mouse was corrected to its body weight.

Analysis of covariance (ANCOVA) was done using the *car*packaging using R (v 4.0.3.). Assumptions of independence between covariates (body weight) and the treatment variables (genotype and diet) were tested using an analysis of variance (ANOVA) model (p > 0.05) and homogeneity of variance was testing using a Levene’s test in the *car* package, (p > 0.05). The type III sum of squares ANCOVA tested the impact of genotype (WT vs KO) and diet (CD vs CR) on energy expenditure, to control for the confounding impact of weight on energy expenditure, we used body weight as a covariate (model: Energy Expenditure ~ Genotype + Diet + Body Weight).

**QUANTIFICATION AND STATISTICAL ANALYSIS**

Lifespan was assessed using the log-rank test by comparing the differences in Kaplan-Meier survival curves (Mitchell *et al.*, 2019). Maximum lifespan was assessed by the average lifespan of the top 10% longest lived mice and Boschloo's Test. Both Log-rank test and Boschloo’s test were conducted using Online Application for Survival Analysis 2 (OASIS 2) with default settings (Han *et al.*, 2016). Results are plotted as mean ± SEM unless otherwise specified, with *p*≤0.05 considered statistically significant. Outliers were excluded using ROUT method with Q = 5%. Data were analyzed by one- or two-way ANOVA followed by t-test. *p* values reported for each comparison were corrected by ﻿Tukey HSD post-hoc test when it is applicable. Significant (p≤0.05) diet effect, genotype effect and/or diet and genotype interaction for each experiment are indicated in figures. Metabolite levels and gene expression were analyzed by unpaired t-test with *p* value uncorrected for multiple comparisons. Analyses and visualization were performed using Graph Pad Prism (9.0.0, GraphPad Software), Excel (Version 16.52, Microsoft), RStudio (Version 1.4.1106), Matlab (R2008a MatLab, MathWork).

**REFERENCES:**

Agrawal, S. *et al.* (2019) ‘EL-MAVEN: A fast, robust, and user-friendly mass spectrometry data processing engine for metabolomics’, in *Methods in Molecular Biology*. Methods Mol Biol, pp. 301–321. doi: 10.1007/978-1-4939-9236-2_19.

Baeza, J. *et al.* (2020) ‘Revealing Dynamic Protein Acetylation across Subcellular Compartments’, *Journal of Proteome Research*. American Chemical Society, 19(6), pp. 2404–2418. doi: 10.1021/acs.jproteome.0c00088.

Biesiadecki, B. J. *et al.* (1999) ‘A Gravimetric Method for the Measurement of Total Spontaneous Activity in Rats’, *Proceedings of the Society for Experimental Biology and Medicine*. Proc Soc Exp Biol Med, 222(1), pp. 65–69. doi: 10.1111/j.1525-1373.1999.09996.x.

Bruss, M. D. *et al.* (2010) ‘Calorie restriction increases fatty acid synthesis and whole body fat oxidation rates’, *American Journal of Physiology-Endocrinology and Metabolism*. American Physiological Society Bethesda, MD, 298(1), pp. E108–E116. doi: 10.1152/ajpendo.00524.2009.

Doerrier, C. *et al.* (2018) ‘High-resolution fluorespirometry and oxphos protocols for human cells, permeabilized fibers from small biopsies of muscle, and isolated mitochondria’, in *Methods in Molecular Biology*. Methods Mol Biol, pp. 31–70. doi: 10.1007/978-1-4939-7831-1_3.

Han, S. K. *et al.* (2016) ‘OASIS 2: online application for survival analysis 2 with features for the analysis of maximal lifespan and healthspan in aging research’, *Oncotarget*. Impact Journals, 7(35), pp. 56147–56152. doi: 10.18632/ONCOTARGET.11269.

Haws, S. A. *et al.* (2020) ‘Methyl-Metabolite Depletion Elicits Adaptive Responses to Support Heterochromatin Stability and Epigenetic Persistence’, *Molecular Cell*, 78(2), pp. 210-223.e8. doi: 10.1016/j.molcel.2020.03.004.

Hepple, R. T. *et al.* (2005) ‘Long‐term caloric restriction abrogates the age‐related decline in skeletal muscle aerobic function’, *The FASEB Journal*. John Wiley & Sons, Ltd, 19(10), pp. 1320–1322. doi: 10.1096/fj.04-3535fje.

Hofer, T. *et al.* (2006) ‘A method to determine RNA and DNA oxidation simultaneously by HPLC-ECD: Greater RNA than DNA oxidation in rat liver after doxorubicin administration’, *Biological Chemistry*. Biol Chem, 387(1), pp. 103–111. doi: 10.1515/BC.2006.014.

Kuznetsov, A. V *et al.* (2008) ‘Analysis of mitochondrial function in situ in permeabilized muscle fibers, tissues and cells’, *Nature Protocols*. Nature Publishing Group, 3(6), pp. 965–976. doi: 10.1038/nprot.2008.61.

Latorre-Muro, P. *et al.* (2018) ‘Dynamic Acetylation of Phosphoenolpyruvate Carboxykinase Toggles Enzyme Activity between Gluconeogenic and Anaplerotic Reactions’, *Molecular Cell*. Elsevier, 71(5), pp. 718-732.e9. doi: 10.1016/j.molcel.2018.07.031.

Mitchell, S. J. *et al.* (2019) ‘Daily Fasting Improves Health and Survival in Male Mice Independent of Diet Composition and Calories’, *Cell Metabolism*, 29, pp. 221–228. doi: 10.1016/j.cmet.2018.08.011.

Patil, Y. N. *et al.* (2015) ‘Cellular and molecular remodeling of inguinal adipose tissue mitochondria by dietary methionine restriction’, *Journal of Nutritional Biochemistry*. J Nutr Biochem, 26(11), pp. 1235–1247. doi: 10.1016/j.jnutbio.2015.05.016.

Pugh, T. D., Klopp, R. G. and Weindruch, R. (1999) ‘Controlling caloric consumption: Protocols for rodents and rhesus monkeys’, *Neurobiology of Aging*, 20(2), pp. 157–165. doi: 10.1016/S0197-4580(99)00043-3.

Scariot, P. P. M. *et al.* (2016) ‘Continuous aerobic training in individualized intensity avoids spontaneous physical activity decline and improves MCT1 expression in oxidative muscle of swimming rats’, *Frontiers in Physiology*. Front Physiol, 7(APR). doi: 10.3389/fphys.2016.00132.

Scariot, P. P. M. *et al.* (2019) ‘Housing conditions modulate spontaneous physical activity, feeding behavior, aerobic running capacity and adiposity in C57BL/6J mice’, *Hormones and Behavior*. Elsevier, 115(July), p. 104556. doi: 10.1016/j.yhbeh.2019.07.004.

Schmiedl, A. *et al.* (1990) ‘The surface to volume ratio of mitochondria, a suitable parameter for evaluating mitochondrial swelling - Correlations during the course of myocardial global ischaemia’, *Virchows Archiv A Pathological Anatomy and Histopathology*. Virchows Arch A Pathol Anat Histopathol, 416(4), pp. 305–315. doi: 10.1007/BF01605291.

Ullman-Culleré, M. H. and Foltz, C. J. (1999) ‘Body condition scoring: A rapid and accurate method for assessing health status in mice’, *Laboratory Animal Science*, 49(3), pp. 319–323.
