# Supplementary material for: SIRT3 deficiency decreases oxidative metabolism capacity but increases lifespan in male mice under caloric restriction
Source: Aging Cell. 2022 Oct 5;21(12):e13721. doi: 10.1111/acel.13721 (PMC9741511; doi:10.1111/acel.13721)
Supplement: Supplementary file 3 — Table S1 [file ACEL-21-e13721-s004.pdf]

Table S1.

A. Log-rank test-based lifespan statistics

Treatment	Number of subjects	Average lifespan (95% CI) months	Age in months at % mortality					Log-rank test	
			25%	Median (50%)	75%	90%	100%	Comparisons	*p*-value
WTCD	45	24.08 (22.17 ~ 26.00)	20.57	25.2	27.3	31.57	39.2	WTCD vs. *Sirt3*$^{-/-}$CD	0.6378
Sirt3$^{-/-}$CD	59	23.46 (21.61 ~ 25.32)	19.43	23.97	28.4	33.9	37.9	WTCR vs. *Sirt3*$^{-/-}$CR	0.0356
WTCR	61	29.65 (28.00 ~ 31.29)	25.73	30.13	34.27	36.67	44.37	WTCD vs. WTCR	0.0001
Sirt3$^{-/-}$CR	75	30.91 (29.04 ~ 32.77)	24.93	31.5	37.7	41.13	46.47	*Sirt3*$^{-/-}$CD vs. *Sirt3*$^{-/-}$CR	8.90E-09

B. Boschloo's test-based lifespan statistics

Comparisons	*p* value at 25% percentile	*p* value at 50% percentile	*p* value at 75% percentile	*p* value at 90% percentile
WTCD vs. *Sirt3*$^{-/-}$CD	0.5534	0.278	0.8318	0.4728
WTCD vs. WTCR	0.0046	0.00001	0.0004	0.1427
WTCD vs. *Sirt3*$^{-/-}$CR	0.0084	4.1E-06	0.0003	0.038
Sirt3$^{-/-}$CD vs. WTCR	0.0022	1.6E-06	0.0076	0.0385
Sirt3$^{-/-}$CD vs. *Sirt3*$^{-/-}$CR	0.0079	4.4E-06	0.0001	0.0095
WTCR vs. *Sirt3*$^{-/-}$CR	0.8704	0.5316	0.0268	0.0405

Table S1.
A) Log-rank test-based lifespan statistics of Kaplan-Meier survival curves shown in Fig.1B.
B) Boschloo's test (Wang-Allison)-based lifespan statistics of Kaplan-Meier survival curves shown in Fig.1B.
Both Log-rank test and Boschloo's test were calculated using Online Application for Survival Analysis 2 (OASIS 2) (Han *et al.*, 2016).
